# Evidence for the rapid expansion of microRNA-mediated regulation in early land plant evolution

**DOI:** 10.1186/1471-2229-7-13

**Published:** 2007-03-14

**Authors:** Isam Fattash, Björn Voß, Ralf Reski, Wolfgang R Hess, Wolfgang Frank

**Affiliations:** 1 Faculty of Biology, Institute of Biology II, Plant Biotechnology, University of Freiburg, Schaenzlestrasse 1, 79104 Freiburg, Germany; 2 Faculty of Biology, Institute of Biology II, Experimental Bioinformatics, University of Freiburg, Schaenzlestrasse 1, 79104 Freiburg, Germany

## Abstract

**Background:**

MicroRNAs (miRNAs) are regulatory RNA molecules that are specified by their mode of action, the structure of primary transcripts, and their typical size of 20–24 nucleotides. Frequently, not only single miRNAs but whole families of closely related miRNAs have been found in animals and plants. Some families are widely conserved among different plant taxa. Hence, it is evident that these conserved miRNAs are of ancient origin and indicate essential functions that have been preserved over long evolutionary time scales. In contrast, other miRNAs seem to be species-specific and consequently must possess very distinct functions. Thus, the analysis of an early-branching species provides a window into the early evolution of fundamental regulatory processes in plants.

**Results:**

Based on a combined experimental-computational approach, we report on the identification of 48 novel miRNAs and their putative targets in the moss *Physcomitrella patens*. From these, 18 miRNAs and two targets were verified in independent experiments. As a result of our study, the number of known miRNAs in *Physcomitrella *has been raised to 78. Functional assignments to mRNAs targeted by these miRNAs revealed a bias towards genes that are involved in regulation, cell wall biosynthesis and defense. Eight miRNAs were detected with different expression in protonema and gametophore tissue. The miRNAs 1–50 and 2–51 are located on a shared precursor that are separated by only one nucleotide and become processed in a tissue-specific way.

**Conclusion:**

Our data provide evidence for a surprisingly diverse and complex miRNA population in *Physcomitrella*. Thus, the number and function of miRNAs must have significantly expanded during the evolution of early land plants. As we have described here within, the coupled maturation of two miRNAs from a shared precursor has not been previously identified in plants.

## Background

MicroRNAs (miRNAs) are highly specific regulators of gene expression. Their target mRNAs become recognized through short stretches of partial complementarity [1]. Upon binding, the mRNA is either cleaved at a distinct site of the miRNA-mRNA duplex or its translation becomes inhibited [1-3]. This phenomenon, which is known as posttranscriptional gene silencing, was first identified in *C. elegans *[4], but was soon shown to be a regulatory mechanism in plants and animals. MiRNA precursors possess a very characteristic secondary structure. This structure consists of a terminal hairpin loop and a long stem [1,3,5] in which the miRNA is positioned [6-8]. The investigation of miRNA biogenesis pathways revealed components that are common to plants and animals, but considerable divergence also exists [9-12]. Their genes are transcribed by RNA polymerase II [13-15], occasionally in the form of di- or even polycistronic primary transcripts [7,16-18]. The maturation of miRNA primary transcripts (pri-miRNAs) differs in plants and animals. In animals, the pri-miRNAs are processed in the nucleus by the microprocessor complex containing the enzyme Drosha and its cofactor, the protein DGCR8 (in humans), or Pasha (in *Drosophila *and *C. elegans*) [19-21]. As a result, ~60–70 nt miRNA precursors (pre-miRNA) are released, which are then exported to the cytoplasm by the nuclear transport receptor exportin-5 [22]. The final maturation step is mediated in the cytosol by Dicer, resulting in a complex between the ~22 nt miRNA and its complementary fragment, miRNA* [23,24]. In plants, homologs of Drosha or its cofactors could not be identified. Furthermore, in *Arabidopsis *the Dicer-like protein 1 is a nuclear protein suggesting that maturation of miRNAs in plants occurs in the nucleus. HASTY is the most likely candidate for a plant homolog of the nuclear transport receptor exportin-5 [25]. However, additional miRNA export mechanisms may exist in plants as *hasty *mutants showed a decreased accumulation of some, but not all miRNAs [25].

Several studies have addressed the composition of the miRNA pool in plants and animals. These studies have been accomplished through shot-gun sequencing of cDNAs obtained from size-fractionated RNA samples, computational prediction from genomic data, or a combination of both [26]. Exploiting their typical stem-loop structure, a large number of computational precursor predictions have been performed [1,27-34]. Recently, a new algorithm was developed to predict miRNAs and their genes based on sequence conservation. This algorithm was successfully used for the prediction of miRNA families conserved among different plant species [35]. These reports support that, like in animals, particular miRNA families are conserved across all major plant lineages and frequently control the expression of mRNAs encoding proteins of the same family [36-38]. Thus, regulatory effects mediated through such miRNAs are likely to be conserved throughout the plant radiation and must have originated anciently. However, it was also demonstrated that certain miRNAs are species-specific [18]. Thus, without the identification of all the miRNAs present in plants at key phylogenetic positions, the evolutionary dynamics of plant miRNAs and their biological functions will not be understood. Similar studies of this type in the animal field suggested the expansion of specific miRNA sets during key transitions in animal evolution [39]. An important evolutionary transition in the plant kingdom occurred when they began life on land. Plants very similar to the first photosynthetic organisms which successfully colonized the land approximately 450 million years ago [40], the Bryophytes (mosses), still exist today. Compared to animal evolution, this time would relate to the evolutionary distance between fish and mammals. However, the transition from an aquatic to a terrestrial lifestyle in plants required far more adaptations than in the mammals-fish example. This transition would have been less complicated for mammals-fish since all major vertebrate cell types and organs were already present in fish. On the contrary, the evolution from green algae towards land plants required the invention of almost all plant organs that are typical for a land-bound lifestyle. The rapid development of many new cell types, organs and adaptations that occurred during early evolution of mosses must have been coupled to an explosive diversification of old genes and the development of new genes [41-43]. It is reasonable to assume that this genetic diversification was paralleled by an equally rapid amplification of new regulatory mechanisms, including miRNAs [44]. Indeed, not a single miRNA has been found so far in genome projects targeting green algae, the immediate evolutionary precursors of land plants [45]. Only few reports have dealt with the analysis of moss miRNAs so far [18,36,37]. Analyzing EST sequences from a large number of plant species, including the moss *Physcomitrella patens*, Zhang et al. [18] identified two conserved miRNAs. The most comprehensive miRNA analysis in *Physcomitrella *so far identified 30 individual miRNAs by cloning. Eleven of these 30 miRNAs belong to four conserved plant miRNA families, whereas the remaining 19 miRNAs had not been previously identified in other plants [17,46]. Recently, large scale pyrosequencing suggested the presence of a larger number of miRNAs in *Physcomitrella *but these were not further characterized [47]. Thus, the knowledge on moss miRNAs is restricted to a small number of studies so far, but these have clearly indicated that some miRNAs evolved in this group before the diversification of land plants.

Until now, a genome-wide analysis of miRNAs was impossible due to the lack of comprehensive genomic sequence information for any moss species. *Physcomitrella patens *has become a valuable model species based on its unique ability to integrate DNA into its nuclear genome by homologous recombination, thereby enabling rapid functional analyses by reverse genetics [48,49]. To further extend its use as a model organism, a genome project has been recently launched. The *Physcomitrella *genome represents the fourth fully sequenced land plant genome in addition to those of *Arabidopsis*, rice and poplar and it is the first one of a non-seed plant. The genome assembly is still underway; however, the WGS traces have been made publicly available.

Here, we report the identification of 48 novel *Physcomitrella *microRNAs through a combined experimental-computational approach. In the computational section we scanned the genomic traces as well as the most comprehensive *Physcomitrella *EST databases [41,42,50] for their precursors and identified 59 potential target mRNAs. The majority of these mRNAs encode several transcription factors, cyclophilins, redox catalysts, enzymes involved in producing the complex cell wall polysaccharides on the plant surface, or other proteins involved in signal transduction processes, such as heterotrimeric G proteins, histidine kinases or factors for alternative splicing. Thus, the functional annotation of target genes revealed a bias towards regulation, signal transduction, cell wall biosynthesis and defense.

We observed the tissue-specific maturation of one miRNA from a precursor also containing another miRNA, a situation not found in plants so far. A comparison of the *Physcomitrella *miRNA families to those of other plants increased the number of miRNA families with a common ancient origin to 17 and identified 18 moss-specific miRNA families. The data indicate an explosion of miRNA diversity and functional diversification which occurred at a key evolutionary transition early in land plant evolution.

## Results

### Cloning of miRNAs from *Physcomitrella patens*

**Figure 1:**
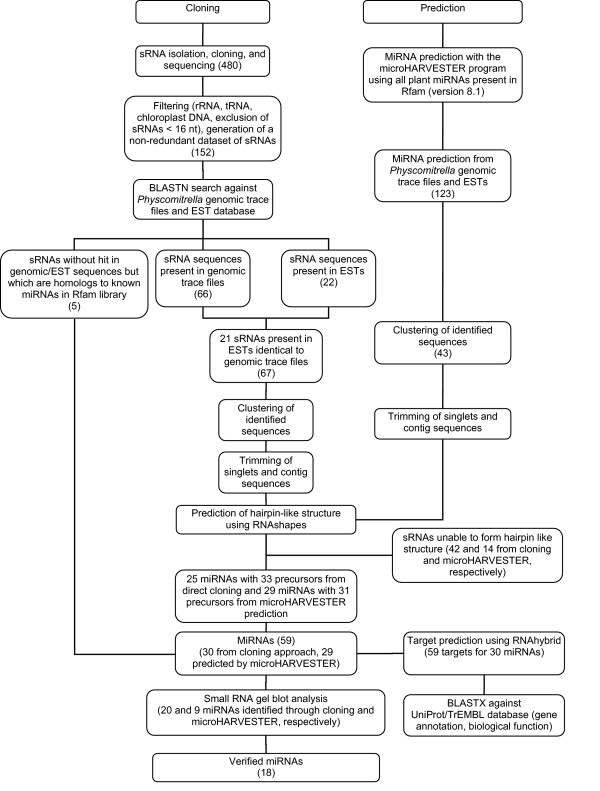
**Schematic presentation of miRNA identification in *Physcomitrella***. MicroRNAs from *Physcomitrella *were identified by cloning of sRNAs and computational prediction using the microHARVESTER program. The flowchart depicts the consecutive filtering and analytical steps applied during miRNA identification.

**Figure 2:**
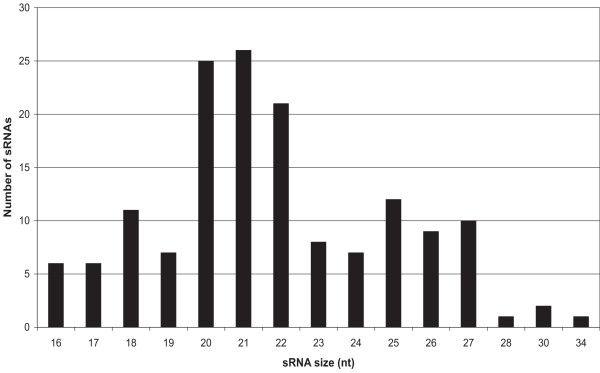
Size distribution of cloned *Physcomitrella *sRNAs.

**Figure 3:**
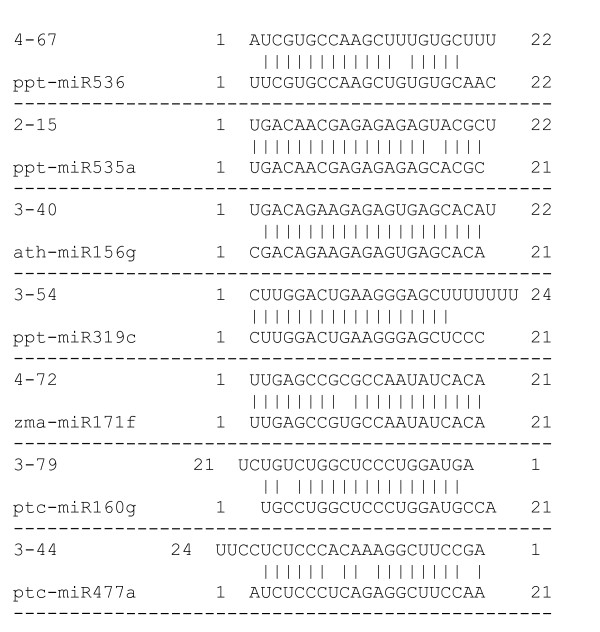
Sequence alignment of cloned miRNAs and previously reported homologous plant miRNAs.

It has been reported that the expression of plant miRNAs may be regulated in a tissue-specific manner [9,51]. Therefore, RNA was prepared from the juvenile *Physcomitrella *protonema as well as the leafy gametophores [52] to cover these two different developmental stages. The fraction of small RNAs of ~15 to 35 nt were cloned, and 480 randomly chosen cDNA clones were sequenced. Sequences shorter than 16 nt were removed from the initial set, leaving 290 sRNAs for further analysis. These sequences were subjected to serial filtering steps (Figure 1) to remove contaminating sequences. BLAST searches in the Genbank and Rfam databases indicated that 138 sequences (47%) had originated from rRNAs, tRNAs and chloroplast RNAs. These sequences were excluded, resulting in a final set of 152 sRNA sequences for further analysis [see Additional file 1]. 106 sequences (70%) ranged between 19 and 25 nt in size, and among these, the majority had a size of 21 nt (Figure 2). Thus, the size distribution of the cloned sRNAs is in agreement with most known plant miRNAs [46]. Only nine sRNA sequences were obtained more than once [see Additional file 1], indicating both a low redundancy of the generated sRNA library as well as a surprisingly high diversity of the original RNA population. The set of 152 non redundant sequences was compared to the Rfam database (version 8.1) to identify already known miRNAs from *Physcomitrella *and other plant species. Six different miRNAs, 2–86, 4–34, 2–31, 2–88, 3–60, and 5–33, were identical to the previously described *Physcomitrella *miRNAs miR1218, miR1212, miR535, miR156, miR536, and miR537, respectively [17,46]. Five sRNAs showed significant similarity to known plant miRNAs and most likely represent additional members of these miRNA families (Figure 3). These sRNAs (4–67, 2–15, 3–40, 3–54) belong to miRNA families miR536, miR535, miR156 and miR319 previously identified in *Physcomitrella *[17,46], whereas the sRNA 4–72 was nearly identical to miR171 present in several other plant species [53]. Thus, among our final set of 152 sRNAs we found only ten miRNAs that were identical or highly similar to one of the 30 previously detected *Physcomitrella *miRNAs. This fact confirms that a surprisingly diverse and complex miRNA population exists in moss. Intriguingly, we also identified two sRNAs, 3–79 and 3–44, which resemble the nearly identical reverse complementary sequences of the known miRNAs miR160 and miR477 [31] (Figure 3).

### Identification of stem-loop precursors of cloned sRNAs

One essential feature of transcripts originating from miRNA-coding genes is their characteristic stem-loop structure. For the further characterization of the cloned sRNAs, we searched for putative miRNA precursors within the genomic trace file archive and EST databases. All sequences containing an sRNA-identical nucleotide pattern were clustered to generate a non-redundant set of putative precursors (compare Figure 1). Furthermore, jointly clustered genomic and EST sequences with identity to the same sRNA were aligned with each other to reveal if the EST sequence represented the transcript of the respective genomic region. For 67 cloned sRNAs, at least one sequence was identified in the genomic traces and/or in the EST database with a perfect sequence match. Within this set, we identified 22 EST sequences and 21 out of these were found to be identical to genomic sequences. These data suggest that they are the unprocessed transcripts of these genomic regions. All clustered sequences were subjected to a precursor analysis based on secondary structure. The structure prediction revealed that 33 sequences encoding 25 of the cloned sRNAs were able to form a hairpin-like structure (Table 1) [see Additional file 2]. In one case (2–70), a putative precursor sequence was only found in the EST database. The identification of these RNAs by cloning, together with the existence of corresponding precursor sequences, suggests that these sRNAs are, in fact, miRNAs from *Physcomitrella*. For five sRNA sequences (2–15, 3–40, 3–44, 3–54, 3–79), no precursors were found whereas their sequences showed significant similarity to plant miRNA families present in Rfam (Figure 3). Therefore, we considered these sequences to be miRNAs as well. The failure to detect identical sequences in the genomic or EST databases could be due to their unfinished status or insufficient coverage. Taken together, the cloning approach led to the identification of 31 miRNAs among the 152 non-redundant sRNAs. Even by the most conservative criteria, 25 miRNAs have not been previously identified in *Physcomitrella*. Among these, 17 cloned miRNAs seem to be species-specific for *Physcomitrella *whereas the remaining eight miRNAs most likely represent new members of conserved plant miRNA families (Table 1). Seven miRNAs (1–63, 2–31, 2–88, 3–60, 5–21, 4–66, 4–72) might be derived from more than one genomic locus as two to three genomic sequence clusters fulfilled the structural requirements of miRNA precursors. In contrast, 18 miRNAs (Table 1) could derive from single copy genes as only one genomic sequence cluster was found for each of these miRNAs. However, this calculation might be an underestimation considering the unfinished character of the *Physcomitrella *genome sequence.

In regards to the maturation pathways of miRNAs, the prediction of genomic precursors revealed some interesting aspects of the miRNAs within this study. The two miRNAs 1–50 and 2–51 are located side by side within the 5' arm of the predicted precursor, and separated by only one nucleotide. Thus, they are very likely processed from a common precursor transcript. miRNAs 1–63 and 3–14 exhibit nearly completely reverse complementarity to each other and are possibly derived from the same precursor [see Additional file 2]. Thus, they might be a pair of miRNA and miRNA*. However, for miRNA 1–63 another, specific precursor was identified [see Additional file 2].

### Prediction of miRNA homologs in *Physcomitrella*

Genomic trace files and EST sequences from *Physcomitrella *were examined for all plant miRNAs present in miRBase (version 8.1) using microHARVESTER [35]. The identified genomic, as well as EST sequences, which were able to form stable hairpin-like structures were further analyzed manually. In total, a redundant set of 123 possible miRNA precursor sequences was generated by microHARVESTER. To obtain a non-redundant set of putative miRNA precursors, all genomic and EST precursor sequences were merged, clustered and further analyzed with RNAshapes [54], applying the same parameters which were previously used for the cloned sRNAs. This analysis revealed 31 sequences producing stable hairpin-like precursor structures encoding 29 individual miRNAs which were assigned to 19 plant miRNA families (Table 2) [see Additional file 2]. Five of these miRNAs were previously described in *Physcomitrella *[17,46], whereas the remaining 24 miRNAs are new for *Physcomitrella *but share high similarities to miRNAs from other plants. Two miRNAs (miR390-2, miR477) seem to have more than one precursor in the genomic or EST sequences set (Table 2) [see Additional file 2].

The *Physcomitrella *miRNA sequences obtained by cloning and bioinformatic prediction were deposited in miRBase [55] [see Additional file 3].

### Detection of *Physcomitrella *miRNAs by small RNA gel blots

**Figure 4:**
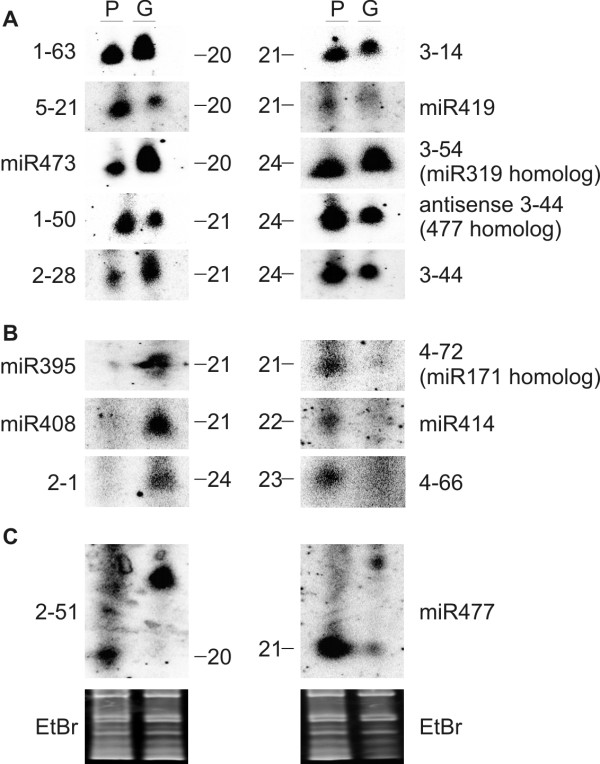
**Detection of miRNAs by small RNA gel blot hybridisation**. *(A) Physcomitrella *miRNAs expressed in protonema (P) and gametophore (G) tissue. *(B) Physcomitrella *miRNAs with a tissue-specific expression pattern. *(C) *Tissue-specific processing of miRNA precursors. The mature miRNAs were detected in RNA derived from protonema tissue, longer incompletely processed precursor transcripts were present in RNA from gametophores. The lowermost panel shows two representative ethidium bromide stained gels to indicate equal loading of the RNAs.

To obtain genuine proof for the presence of miRNAs which were identified by cloning or computational analysis, a set of 29 miRNAs (20 from cloning, 9 from prediction) was chosen for expression analysis by small RNA gel blots. As the cloned miRNAs were derived from protonema and gametophores, total RNA from these tissues was used for RNA gel blot preparation. Among the selected miRNAs, we chose four putative miRNAs for which no possible precursors had been identified in the genomic traces and EST sequences, but which show high similarity to known miRNAs. Twelve miRNAs which were identified by the cloning approach and six miRNAs which were computationally predicted were detected by gel blot hybridization (Figure 4, Tables 1 and 2). No signals were found for the remaining 11 miRNAs, probably a consequence of their low expression level. Yet, these sRNAs are still considered to be miRNAs. We conclude this since stem-loop containing precursors were predicted, the characteristic diagnostic feature for this class of sRNAs, and because 8 of these 11 miRNAs (1–22, 1–39, 3–5, 3–62, 3–91, 4–12, 2–70, 3–79) had been found by cloning. Ten miRNAs (1–63, 5–21, miR473, 1–50, 2–28, 3–14, miR419, 3–54, 3–44, 3–44 antisense) were detected in both protonema and gametophore tissue in nearly equivalent amounts. Interestingly, the miRNA 1–63 and its nearly identical reverse complement counterpart 3–14, were both detected with high abundance. These data indicate that these are *bona fide *miRNAs rather than representing miRNA/miRNA* (see above). The cloned sRNA 3–44 was nearly an identical reverse complement sequence of the previously published miR477. However, 3–44 is 24 nt in size whereas miR477 has a length of 21 nt [31]. Hybridization with strand-specific probes revealed that 3–44, as well as its complementary RNA (3–44-antisense), accumulated in almost equal amounts in both protonema and gametophore tissue, both with an identical length of 24 nt. Thus, these two RNAs possibly constitute a case of co-accumulating miRNA/miRNA*. Moreover, we also detected the 21 nt miR477 in our expression studies revealing the existence of highly similar miRNAs which only vary in size.

### Tissue-specific expression of miRNAs

Three miRNAs (miR414, 4–72, 4–66) were exclusively expressed in protonema, whereas another three miRNAs (miR395, miR408, 2-1) were detected only in gametophores, thereby indicating tissue-specific expression of these miRNAs.

The precursor prediction suggested that miRNAs 1–50 and 2–51 are transcribed in a shared precursor, separated only by one nucleotide from each other. The expression analysis verified the existence of both miRNAs, but their level and the maturation from the shared precursor varied. MiRNA 1–50 was present in protonema and gametophores, whereas the mature miRNA 2–51 only accumulated in protonema tissue. For miRNA 2–51, however, a signal for a larger RNA molecule of approximately 60 nt was also detected in gametophores. We assume that this larger RNA fragment represents an incompletely processed precursor transcript. Thus, processing of the two miRNAs 1–50 and 2–51 originating from the same precursor is different in the two analyzed moss tissues. Intriguingly, the two miRNAs 1–50 and 2–51 have no homologs in mirBase and are thus considered to be moss-specific. Another case was observed for miR477 (Figure 4), where the mature miRNA was present in protonema and an incompletely processed larger precursor was identified in RNA derived from gametophores.

### Detection of homologs of cloned miRNAs from *Physcomitrella *in other plant species

All *Physcomitrella *miRNAs predicted by micoHarvester exist in other plants as well, since that algorithm solely finds homologs to already known miRNAs. However, up to 17 out of the total of 29 cloned miRNAs could be species-specific as these do not have close homologs in miRBase (version 8.1). This number could be misleading since the database might not be complete. Therefore, an independent screen was implemented in which these species-specific miRNAs were used as query sequences to identify possible homologs in the completely sequenced genomes of *Arabidopsis*, poplar and rice directly using microHARVESTER. For one miRNA, 4–12, a homolog in rice harboring a characteristic stem-loop structure was predicted [see Additional file 4]. Thus, the rice homolog of miRNA 4–12 might have been overlooked in previous analyses and consequently, the miRNA 4–12 was not further regarded as moss-specific.

### Comparison of plant miRNAs

Including the results presented here, the number of known *Physcomitrella *homologs to plant miRNA families has been raised from 4 to 17. The direct comparison of miRNA families which are shared by at least by two different plant species allows new insights into the evolution of plant miRNAs. In order to generate the most comprehensive overview, all plant miRNAs in miRBase were compared with each other and with all *Physcomitrella *miRNAs described here or before [see Additional file 5]. This analysis revealed the existence of 35 plant miRNA families shared by at least two plant species. Eighteen miRNA families seem to be absent in *Physcomitrella *although they are common to most other plant species. For comparison, 24 families have not yet been found in *Glycine maximum*, whereas only three are absent from *Arabidopsis*. These observations indicate that these numbers are heavily influenced by the sampling depth in the different plants.

However, even if interpreted with great caution, the miRNA families 169 and 399 contain numerous individual members in other plants, but seem to be missing in *Physcomitrella *altogether. Thus, these families might have originated after the divergence between those plant lineages and mosses. *Physcomitrella *is underrepresented in some miRNA families, where several members were identified in other plant species, but only one member was found in *Physcomitrella *(e.g. miRNA families 166, 167, 172, 395). Therefore, these families may constitute examples for miRNAs with a common ancient origin followed by amplification in higher plants. In contrast, *Physcomitrella *contains more individual miRNA members in the families 477, 535, 390 and 319. Thus, these miRNA families either have expanded in the moss or their size was reduced during land plant evolution.

During this analysis, we also analyzed the gene copy number for particular miRNAs. Apparently, the majority of *Physcomitrella *miRNAs are encoded by single genes, whereas the identical miRNA in other species is often encoded by more than one gene [see Additional file 5]. Thus, the gene copy number per miRNA has increased during land plant evolution.

### Target prediction

**Figure 5:**
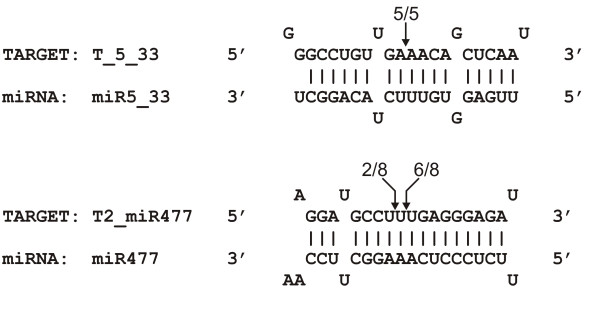
Validation of predicted miRNA targets T_5_33 and T2_miR477. RNA ligase-mediated 5' RACE-PCRs were performed with gene-specific primers and resulting PCR products were sequenced. The sequences depict the miRNA binding site within the target mRNA and numbers above indicate the detected cleavage site of independent clones.

The high complementarity between plant miRNAs and their target genes allows an effective prediction of the target sequences through computational analysis [56-60]. Here, all identified 59 miRNAs, including those previously reported, were used to search the *Physcomitrella *EST database with RNAhybrid [61] for complementary hits. In this analysis we used the parameters developed by Schwab et al. [60] for identifying authentic miRNA targets in plants. This analysis yielded 59 potential target genes for 30 individual miRNAs (Table 3) [see Additional file 6]. The number of targets per miRNA varies widely, from 1 to 12. For 16 out of the 30 miRNAs one target was predicted and seven miRNAs target two mRNAs. The miRNAs 1–63, miR473-2, miR160-2, miR160-3, each target three mRNAs, whereas miR408, miR477, and miR414 have 5, 7, and 12 predicted targets, respectively (Table 3). We have validated the targets T2_miR477 homologous to a CONSTANS-like transcription factor and T_5_33 homologous to a protein of unknown function by RNA ligase-mediated 5' RACE-PCR. The obtained fragments end at the expected sites between nucleotide position 10 and 11 within the miRNA binding site. These data clearly indicate that both mRNAs are in fact targets of miRNAs 477 and 5–33, respectively (Figure 5).

Some of the miRNAs which belong to the same miRNA family most likely regulate the identical target genes, suggesting a functional redundancy of these miRNAs (e.g. 160-1, 160-2, 160-3, 160-4). In contrast, for other miRNA families (171, 319, 533, 534) specific target genes were predicted for the individual family members, indicating a high specificity of the miRNA/target interaction even though the miRNA sequence has been highly conserved within the respective miRNA family. For two miRNAs which belong to different miRNA families (miR473-1 and miR477), one shared target mRNA was identified, indicating that these two different miRNAs regulate the same mRNA. Intriguingly, both miRNAs target the same mRNA region with one nucleotide offset. As suggested for *Arabidopsis*, these miRNAs may have evolved by duplication of target sequences [62,63].

Members of the miRNA160 family control the expression of an auxin response factor homolog in *Physcomitrella *as well as in other plant species [37]. Furthermore, miR166 was predicted to target a class III homeodomain leucine-zipper transcription factor. This prediction is in accordance with previous reports on the miRNA166 family-mediated regulation of this class of transcription factors in all lineages of land plants [36]. Additionally, the identified *Physcomitrella *miR408 and miR477 seem to control conserved target genes previously predicted in *Populus trichocarpa *[59,64].

In fact, the individual analysis revealed a strong bias among the predicted target mRNAs (Table 3). A large number (21) of predicted targets are involved in regulation, e.g. transcription factors or signal transduction proteins. The second largest group (19) of targets consists of mRNAs without a known function or for which no reasonable homologs exist in the public databases. Interestingly, twelve targets can be related to adaptations to life on land, such as the formation of cell wall and defense (3 and 9 targets, respectively). One example is the target T_1-39 of miRNA 1–39 coding for a mucin-like protein as its closest homolog. Mucins carry a dense sugar coating which provides considerable water-holding capacity and also makes them resistant to proteolysis. Of the remaining targets, eight are metabolism-associated. Among these, two mRNAs encode proteins involved in sulfur metabolism. *Physcomitrella *uses more diverse routes of sulfate assimilation than angiosperms [65], thus a need for their specific regulation through miRNAs is likely. Another notable target is T_5-21 which is related to 2S albumin, a plant seed protein, which might accumulate in a homologous fashion solely in moss spores and thus needs to be down-regulated in all other tissues. For some predicted targets, e.g. cyclophilins and F-Box proteins, it is known or at least predicted that their homologues in other plants or even in vertebrates underlie miRNA regulation as well (see Table 3 for comments). These targets have no direct sequence similarity to *Physcomitrella*, indicating their independent origin through convergent evolution, or too large divergence accumulated over long evolutionary time scales.

These findings, and the conservation of miRNA/target-pairs described before, provide further evidence that particular miRNAs and their corresponding targets must have evolved early in land plant evolution and were then conserved widely throughout the plant radiation.

## Discussion

### Cataloging *Physcomitrella *miRNAs

In our study, we analyzed 152 sRNAs obtained by cloning. After stringent filtering steps, we identified 24 new and six previously known *Physcomitrella *miRNAs among them. Additionally, we used a computational strategy by which 29 individual miRNAs were predicted based on sequence similarity; only five of these had been previously reported from *Physcomitrella *[17,46]. From this collective group of 59 miRNAs, we experimentally validated 18 novel miRNAs. This validation included eight miRNAs specific for *Physcomitrella *and ten homologs of known plant miRNA families. These 18 miRNAs were identified by cloning (12) or prediction (6), indicating a high degree of true positives in the dataset presented in this study. The small overlap in the number of miRNAs found by cloning and through the computational strategy indicates that a combined approach is much more likely to yield a comprehensive set of miRNAs, especially if knowledge about miRNAs in related organisms is available.

Together with the 30 previously reported miRNAs [17,46], we extended the number of known miRNAs in *Physcomitrella patens *to 78. Compared to maize, *Arabidopsis*, rice and poplar where 96, 118, 182 and 213 miRNAs were described, respectively, this number seems small. Hence, it is in good agreement with the idea that a less complex organism than higher plants, gymno- and angiosperms, such as moss, might utilize a less complex set of miRNAs. However, one of the most striking results of this study is that our screen was in no way exhaustive: the vast majority of miRNAs was found only in single copies in our sRNA library and the overlap is about only one third each between the miRNA populations identified by cloning, by computational prediction, or which had been described before. Thus, it is very likely that the number of miRNAs in *Physcomitrella *is much greater than 78 and will well reach numbers known for higher plants. Furthermore, compared to the relatively low number of two *Physcomitrella *miRNAs identified by the analysis of available EST data [18], our investigation of the genomic sequence resulted in a far greater number of miRNAs as presented in this study.

### Tissue-specific maturation of miRNAs as a new level of regulation

In our analyses, we found evidence for unknown processing or maturation steps that have not been previously described and at least eight cases of tissue-specific expression. For miR477 and 2–51, the regulation is achieved posttranscriptionally by tissue-dependent maturation. The most interesting observation is the evidence for the coupled maturation of two miRNAs from a shared precursor. These two miRNAs, 1–50 and 2–51, are located on the same precursor and are separated by only one nucleotide. This presents the first example for plants, as well as for animals, that two miRNAs are processed from the same stem-loop precursor where they reside in close vicinity. Furthermore, the maturation of these two miRNAs from their shared precursor differed between protonema and gametophores. The mature miRNA 1–51 was detected in protonema and gametophores, whereas the mature miRNA 2–51 was only present in protonema. The unprocessed precursor still harboring miRNA 2–51 accumulated in gametophores. Tissue-specific processing of miRNAs as a new level of regulation has not been observed in plants before, while it has been reported for mammals [66]. In consequence, the cleavage of stem-loop precursors of particular miRNAs by Dicer could be restricted to specific cell types or involve additional factors which regulate this specificity. Moreover, the differential processing presents an additional way to control miRNA action besides the tissue-specific transcription of the precursor.

### Evidence for co-accumulating miRNA/miRNA* pairs?

The cloning of miRNAs and their cognate miRNA* has been reported in the literature [6,67,68]. The miRNA* has always been found to be less abundant than the respective miRNA. It has been suggested that after cleavage of the precursor by Dicer, the miRNA becomes part of the RNA induced silencing complex (RISC) whereas its counterpart miRNA* is rapidly degraded [8]. In all cases observed here, the possible miRNA/miRNA* partners were present in comparable amounts. The miRNAs 1–63/3–14 are a potential miRNA/miRNA* pair since they are located on opposite arms of the same precursor and are able to base-pair at least partially with each other. We verified the existence of both miRNAs experimentally. In addition, putative targets for both miRNAs were predicted from the EST sequences, suggesting that both act as miRNAs rather than constituting miRNA/miRNA*. Since a separate precursor was found for miRNA 1–63, the potentially shared 1–63/3–14 precursor may not actually deliver mature 1–63, but this possibility cannot be totally exluded. Another pair of miRNA/miRNA* is represented by miRNAs 3–44 and 3-44-antisense and again, the existence of both miRNAs was experimentally validated. At this stage, however, the final proof for a miRNA/miRNA* pair cannot be provided as we did not find a possible precursor for either of these miRNAs. Thus, it is impossible to determine whether they stem from the same or different precursors and if their nearly equivalent levels of co-accumulation is due to a slow miRNA* degradation rate in *Physcomitrella*. Interestingly, besides being a potential miRNA*, miRNA 3-44-antisense is a homolog to miR477 sharing considerable sequence identity, even though the *Physcomitrella *miRNA 3-44-antisense is 24 nt in size compared to the 21 nt miR477.

### Evolutionary conservation of plant miRNAs

Based on analyzing the evolutionary conservation of miRNAs throughout several plant species, we identified *Physcomitrella *miRNAs belonging to 17 previously described plant miRNA families. In previous studies, members of the miRNA families 156, 319, 390 and 535 were found in *Physcomitrella *[17,46]. The existence of the miRNA families 160, 166 and 172 in *Physcomitrella *was suggested without experimental evidence and was based on the presence of their putative binding sites in conserved target genes [36-38]. Furthermore, the presence of miR160 was shown in the moss *Polytrichum juniperinum *[37]. In this study, we have identified additional *Physcomitrella *miRNAs belonging to 13 conserved plant miRNA families. In some cases, conserved miRNAs present in *Physcomitrella *also target similar genes as those observed in higher plants. We found that miR160 and miR166 most likely control transcription factors in *Physcomitrella *that are homologous to those already reported from other plants [36,37]. In addition, we identified two new miRNAs, miR408 and miR477, for which homologous targets are also predicted at least in *Populus trichocarpa *[59,64]. This co-conservation lets us conclude that these miRNAs regulate central processes common and essential to all plants, such as developmental processes [37].

Moreover, the detailed target analysis revealed a bias towards regulation, signal transduction, cell wall biosynthesis and defense. These processes must have been relevant for the step from water to land and therefore it might not come as a surprise to find these mRNAs in *Physcomitrella*. However, the dominance of these mRNA classes as miRNA targets is stunning. It does provide support for the development of new eukaryotic organs and tissue types in parallel with the explosive expansion of regulatory mechanisms dependent on RNA as recently predicted [44].

However, for 18 other miRNA families which are shared by at least two different plants, no members were found in *Physcomitrella*. Some of these families may have evolved only after the split between mosses and seed plants. Our results clearly demonstrate the ancient roots of many plant miRNA families, whereas others may have evolved after the split between mosses and seed plants. Similar findings have been reported for animals, where inventions of particular miRNA families correspond to major developmental progress like the advent of vertebrates and mammals [39]. Those conserved miRNA families not found in *Physcomitrella *may present similar innovations of the plant miRNA repertoire which coincide with the advent of vascular plants. However, our results also indicate that a considerable number of miRNA families exist in *Physcomitrella *without any counterpart among higher plants. This observation suggests that these families evolved in the moss after their split from the lineage leading to seed plants or were lost during plant evolution. Hence, this set can be seen as miRNAs that separate *Physcomitrella *from higher plants and they may be involved in processes restricted to mosses.

In one example, the search for plant homologs to *Physcomitrella *miRNAs revealed a rice homolog to miRNA 4–12 that might have been overlooked in previous analyses. Consequently, a deeper analysis of the miRNA repertoire of distantly related plants might help to discover more miRNAs in higher plants. Beside the evolution of certain plant miRNA families, the analysis of *Physcomitrella *miRNAs allows one to draw further conclusions on the diversification of these families. In many cases, *Physcomitrella *seems to have less individual members within a given miRNA family. The lower complexity of miRNA families in *Physcomitrella *suggests that the total number of target genes controlled by these miRNAs might be smaller compared to higher plants. Moreover, the miRNA gene copy number seems to be smaller in *Physcomitrella*. The increased number of genes for one particular miRNA in higher plants might be explained by the demand to regulate miRNA expression in a more diverse manner than in *Physcomitrella*. A larger number of gene copies encoding the same miRNA allows the differential expression of these genes by divergent promoters responding to different signaling pathways. Similar scenarios have been observed in expression analyses of multicopy miRNA gene families in *Arabidopsis *and rice [69]. The higher complexity of seed plants, with a large number of different cell types, may require the distinct expression of particular miRNAs in certain cell types. In contrast, mosses have a simpler body plan that is formed by only few different cell types. Therefore, a lower number of miRNA genes may suffice to meet the requirements of a cell-specific expression.

## Conclusion

The identification of 48 novel miRNAs in the moss *Physcomitrella*, an early-branching plant species, and a comparison of miRNAs among various land plant species revealed a considerable number of miRNA families specific for *Physcomitrella*. Other families were retained during land plant radiation or were found to be specific for higher land plants, and thus, may have evolved after the divergence between vascular plants and mosses. The numbers of miRNAs in some families were expanded in higher plants, possibly reflecting the increased complexity of these species. Novel aspects of miRNA biogenesis were found in the maturation of two individual miRNAs from one shared precursor. This is a novel finding as the miRNAs are located side by side and are not complementary to each other. Furthermore, we found evidence for their tissue-specific maturation, uncoupling the presence of the mature forms of these two miRNAs from each other. Thus, processing of these precursors may present another level of control to generate miRNAs in a tissue-specific manner.

## Methods

### Plant material

*Physcomitrella patens *plants were cultured in a modified liquid Knop medium containing 250 mg l^-1 ^KH_2_PO_4_, 250 mg l^-1 ^KCl, 250 mg l^-1 ^MgSO_4_·7H_2_O, 1000 mg l^-1 ^Ca(NO_3_)_2_, and 12.5 mg l^-1 ^FeSO_4_·(pH 5.8) as described by Reski and Abel [70]. Erlenmeyer flasks containing 400 ml of suspension culture were agitated on a rotary shaker at 120 rpm at 25°C under a light/dark regime of 16/8 h (Philips TLD 25, 50 μM m^-2 ^s^-1^). Liquid cultures were mechanically disrupted every week to maintain the plants in the protonema stage. Gametophore development was induced by transferring protonema tissue to solidified Knop medium [71].

### Cloning of small RNAs (sRNAs)

Prior to the isolation of RNA, protonema and gametophore tissue were mixed at a 1:1 ratio. Total RNA was isolated and sRNAs were cloned as described by Llave et al. [72] with minor modifications. Small RNAs (< 200 nt) were separated on a denaturing 12.5% polyacrylamide gel. The population of sRNAs corresponding to 15–35 nt in size was recovered by passive elution from the gel. Following the poly(A)-tailing with *E. coli *poly(A) polymerase, an RNA adaptor (5'-GAATTCCTCTGGACCTTGGCTGTCACTCAAA-3'; *Eco*RI site is underlined) was ligated to the 5' phosphate of the sRNAs. First strand cDNA synthesis was carried out using an oligo(dT)-linker primer (5'-GGATCCCCTTACGAGACATCGCCCCGC-dT_25_; *Bam*HI site is underlined) with M-MLV-RNase H^- ^reverse transcriptase. The resulting cDNAs were amplified by 17 PCR cycles with primers derived from the adaptor sequences and the cDNAs were directionally cloned into the *Eco*RI and *Bam*HI sites of the pBluescript II SK+ vector. Ligation products were electroporated into TransforMaxTM EC100TM electro-competent cells (Epicentre, Oldendorf, Germany) and plasmid DNA from single colonies were isolated and sequenced.

### Sequence and structure analysis

The obtained sRNA sequences were subjected to different filtering procedures. Small RNA sequences shorter than 16 nt in length were removed and were not subjected to further analysis. To examine the origin of the remaining sRNAs and to detect contaminations of the sRNA library with fragments derived from highly abundant RNAs, a non redundant set was generated and searched against GenBank [73] and Rfam [74]. Small RNAs with 100% identity to tRNA, rRNA, or chloroplast DNA were excluded from further analysis. Homologs to known miRNAs were identified using miRBase [75]. In order to detect corresponding miRNA precursor sequences, all putative miRNA sequences were subsequently analyzed by BLAST searches against the *Physcomitrella *genomic trace files and a *Physcomitrella *EST database [42,50]. Genomic or EST sequences with one or more perfect matches to an individual sRNA sequence were clustered and assembled using the Paracel Transcript Assembler. The parameters for clustering threshold, overlap length and overlap identity were 100 nt, 80 nt and 85%, respectively. The generated contig sequences were analyzed with RNAshapes [54] to predict the secondary structure of miRNA precursors. For this, sequences spanning the putative miRNA site were trimmed in three different combinations upstream and downstream of the putative miRNA sequence: (1) 150 bp upstream and 50 bp downstream, (2) 50 bp upstream and 150 bp downstream, and (3) 150 bp upstream and 150 bp downstream of the miRNA sequence. Genomic trace files can be retrieved from NCBI genomic trace archive [76] and EST sequences are available at *Physcomitrella patens *resource cosmoss [77].

### Identification of homologs to known miRNAs from other plants

Evolutionary conservation of miRNA sequences is a feature which can be used to find miRNAs which are homologous to an already identified miRNA from another plant species. Such a strategy is implemented in the tool microHARVESTER [35] which we used to identify homologs to known miRNAs (miRBase) in genomic trace files and EST sequences allowing a maximum number of eight unpaired nucleotides within the mature miRNA sequence. The sequences of predicted precursors of the miRNA homologs were further analyzed by clustering, trimming, and prediction of hairpin-like structures using the same parameters described above.

### Prediction of miRNA target genes

MicroRNA-specific target genes were predicted for the *Physcomitrella *EST database using RNAhybrid [61]. This was done for both possible orientations as the database contains sequences derived from 5' as well as 3' cDNA ends. The target prediction parameters were used according to Schwab et al. [60]: no mismatch at positions 10 and 11, no more than one mismatch at positions 2–12, no more than two consecutive mismatches downstream of position 13, and at least 72% of free energy compared to a perfectly complementary target. The nucleotide sequences of putative targets were used for BLASTX searches against UniProt and TrEMBL in order to get preliminary gene annotation. EST sequences of the target genes are available at the Pyscomitrella patens resource cosmoss [77].

### Small RNA blots

Total RNA from *Physcomitrella *plants was isolated with TRIzol reagent (Invitrogen, Carlsbad, USA) and separated in a 12% denaturing polyacrylamide gel containing 8.3 M urea in TBE buffer (45 mM tris-borate pH 8.0, 1 mM EDTA). RNA gels were stained for 30 min with ethidium bromide (1 μg/ml in TBE buffer) and de-stained for 30 min in TBE buffer. The RNA was electroblotted to Hybond N^+ ^nylon membranes (Amersham, Freiburg, Germany) for 1 h at 400 mA using a trans-blot transfer cell (Bio-Rad, Hercules, CA, USA) and crosslinked by UV light. Radiolabeled RNA probes were generated using *mir*Vana miRNA probe construction kit (Ambion, Huntingdon, UK) according to the manufacturer's instructions. Prehybridization and hybridization of the blots were carried out in 0.05 M sodium phosphate (pH 7.2), 1 mM EDTA, 6 × SSC, 1 × Denhardt's, 5% SDS. Blots were washed 2–3 times with 2 × SSC, 0.2% SDS and once with 1 × SSC, 0.1% SDS. Blots were hybridized and washed at temperatures 5°C below the T_m _of the oligonucleotide. The membranes were autoradiographed using the Molecular FX phosphoimager (Bio-Rad). Blots were stripped in between hybridizations by washing three times 10 min each with 0.1% SDS at 90°C and exposed overnight to verify complete removal of probe before rehybridization.

### Validation of miRNA targets

RNA ligase-mediated rapid amplification of 5' cDNA ends was carried out using the GeneRacer Kit from Invitrogen (Carlsbad, USA). The GeneRacer RNA 5' primer was directly ligated to pooled RNA isolated from protonema and gametophores without previous phosphatase pyrophosphatase treatments. PCR amplification was performed using the GeneRacer 5' primer and 3' gene specific primers for T_5_33 (5'-AATTCTCTGGTGTGTTGTCGGCGGAGAG-3') and T2_miR477 (5'-CAGTCTCAGTAAAGATGGCGCAGCAGGT-3'). Amplified T2_miR477 product was subjected to nested PCR with 1μl of the initial PCR using the GeneRacer 5' nested primer and a 3' gene specific nested primer (5'-CTCCCTCCAGAGAGCACCGCAAGA-3'). PCR products were gel-purified, cloned and then sequenced.

## Authors' contributions

Isam Fattash performed all experiments, carried out bioinformatic analyses and helped drafting the manuscript, Björn Voß performed bioinformatic analyses and helped to draft the manuscript, Ralf Reski helped to draft the manuscript, Wolfgang R. Hess and Wolfgang Frank designed research and wrote the manuscript. All authors read and approved the final manuscript.

## Supplementary Material

Additional file 1 **Non redundant set of cloned sRNA sequences after filtering**. The table shows the complete non-redundant set of sRNA sequences which have been obtained from the sRNA cloning approach. Only those sRNA sequences are listed which did not show homologies to rRNA, tRNA or chloroplast encoded RNA sequences deposited in Rfam. Click here for file

Additional file 2 **Precursor structures of *Physcomitrella *miRNAs**. Fold back analysis of identified potential precursor sequences. Genomic sequences and EST sequences harboring regions identical to sequences of cloned and predicted sRNAs were trimmed and clustered. The non-redundant set of singlets and contigs was used for structural analysis using the RNAshapes program. The mature miRNA sequences within the precursors are highlighted in red. Click here for file

Additional file 3 **miRBase annotations of identified *Physcomitrella *miRNAs**. The table shows annotations of the identified *Physcomitrella *miRNAs which were deposited in miRBase. Click here for file

Additional file 4 **Precursor of a putative rice homolog of *Physcomitrella *miRNA 4–12**. The reciprocal search with microHARVESTER using all *Physcomitrella *miRNAs without previously found homologs in other plants identified a putative homolog for miRNA 4–12 in rice. The corresponding precursor structure of this rice miRNA is depicted in this figure. Click here for file

Additional file 5 **Comparison of conserved plant miRNAs**. In order to analyze conserved plant miRNA families all *Physcomitrella *miRNAs, as well as all plant miRNAs deposited in miRBase, were compared. miRNA sequences which were at least present in two different plant species are listed and the numbers of corresponding precursor sequences are provided. Click here for file

Additional file 6 **Sequence alignments of *Physcomitrella *miRNAs and their putative targets**. The figure shows all sequence alignments between *Physcomitrella *miRNAs and their putative targets detected in a *Physcomitrella *EST database using the RNAhybrid program. Click here for file

## Figures and Tables

**Table 1:** List of *Physcomitrella *miRNAs identified by cloning.

Name	Sequence 5'→3'	Length (nt)	Homolog to known miRNA	Precursor genomic	Precursor EST	Expression verified
1–22	AUUGGGACUUGUGCUGGGAC	20		1 (gnl|ti|872730449)	n.f.	no
1–39	CGUUUCACGUCGGGUUCACC	20		1 (gnl|ti|713836555)	n.f.	no
1–50	UGGCUGAGUCGAAGGUUGUGC	21		1 (gnl|ti|859644225)	n.f.	yes (P/G)
1–63	UUGCUGUGCACUACUUAGUA	20		2 (gnl|ti|1012878547, gnl|ti|835906822)	1 (PR_1-63/3-14)	yes (P/G)
2-1	GUAGCUUAGCGAGGUGUUGGUA	22		1 (gnl|ti|890552627)	1 (PR_2-1)	yes (G)
2–28	CGCUGUCCAUUCUGAGCAUUG	21		1 (gnl|ti|1010151671)	n.f.	yes (P/G)
2–31^a^	UGACAACGAGAGAGAGCACGC	21	miR535 (ppt, osa)	3 (gnl|ti|1003237208, gnl|ti|756805268, gnl|ti|872833603)	n.f.	n.e.
2–42	GUCAAUUUGGCCGAGUGGUUAAGGC	25		1 (gnl|ti|1000320159)	n.f.	n.e.
2–51	GAGCUUUCUUCGGUCCAAUA	20		1 (gnl|ti|859644225)	n.f.	yes (P)
2–86^a^	CCUUAGAGUCGUAGGCCUCUG	21	miR1218 (ppt)	1 (gnl|ti|774610216)	1 (PR_2-86)	n.e.
2–88^a^	UGACAGAAGAGAGUGAGCAC	20	miR156 (ath, osa, zma, sbi, sof, gma, ptc, ppt)	2 (gnl|ti|850661024, gnl|ti|784299453)	n.f.	n.e.
3–5	UGAUCAAGUGGAAACUCAGCAAA	23		1 (gnl|ti|863031657)	n.f.	no
3–14	GCUAGGCAGUGCACAGCGAUA	21		1 (gnl|ti|1012878547)	1 (PR_1-63/3-14)	yes (P/G)
3–60^a^	UUCGUGCCAAGCUGUGUGCAAC	22	miR536 (ppt)	2 (gnl|ti|890625113, gnl|ti|869792930)	1 (PR_3-60)	n.e.
3–62	AACUGAGAUACAUCGCAAUCG	21		1 (gnl|ti|1029072876)	n.f.	no
3–91	GCUGUGUUCUUGUACCUGGG	20		1 (gnl|ti|831706876)	n.f.	no
5–21	UCUUGUCAAUGUUUAGGGGC	20		2 (gnl|ti|891393071, gnl|ti|836345675)	n.f.	yes (P/G)
5–33^a^	UUGAGGUGUUUCUACAGGCU	20	miR537 (ppt)	1 (gnl|ti|903313912)	n.f.	n.e.
4–12	GGUAAAGUGGCGGCUAGGUUA	21		1 (gnl|ti|890397681)	n.f.	no
4–34^a^	CGUGGGACAGCAUAGAAUGCG	21	miR1212 (ppt, pj)	1 (gnl|ti|713871562)	n.f.	n.e.
4–66	ACGAAGGUCUGCAUCAUAGCCAA	23		2 (gnl|ti|1000325696, gnl|ti|816375179)	1 (PR_4-66)	yes (P)
4–67^b^	AUCGUGCCAAGCUUUGUGCUUU	22	miR536 (ppt)	1 (gnl|ti|713832028)	n.f.	n.e.
4–72^b^	UUGAGCCGCGCCAAUAUCACA	21	miR171 (ath, zma, osa, ptc)	2 (gnl|ti|1023219413, gnl|ti|993696673)	n.f.	yes (P)
3–36	GCUACUUCGGCGGGACAAGAGA	22		1 (gnl|ti|1020603193)	n.f.	n.e.
2–70	GUUGGAAGCCUUCGUGGGA	19		n.f.	1 (PR_2-70)	no
2–15^b^	UGACAACGAGAGAGAGUACGCU	22	miR535 (ppt, osa)	n.f.	n.f.	n.e.
3–40^b^	UGACAGAAGAGAGUGAGCACAU	22	miR156 (ath, gma, mtr, osa ptc, sbi, sof, zma, ppt)	n.f.	n.f.	n.e.
3–44^c^	UCGGAAGCCUUUGUGGGAGAGGAA	24	miR477 (ptc)	n.f.	n.f.	yes (P/G)
3–54^b^	CUUGGACUGAAGGGAGCUUUUUUU	24	miR319 (ath, gma, ppt, ptc, sbi, sof, zma)	n.f.	n.f.	yes (P/G)
3–79^c^	UCAUCCAGGGAGCCAGACAGA	21	miR160 (ath, gma, mtr, ptc, osa, sbi, zma)	n.f.	n.f.	no

^a ^Identical to previously identified miRNA. ^b ^Homologous to known miRNA family, but not identical to individual members of this family. ^c ^The reverse and complementary sequence of the miRNA shows similarity to known miRNAs. ath: *Arabidopsis thaliana*, gma: *Glycine max*, mtr: *Medicago truncatula*, osa: *Oryza sativa*, ptc: *Populus trichocarpa*, ppt: *Physcomitrella patens*, pj: *Polytrichum juniperinum*, sbi: *Sorghum bicolor*, sof: *Saccharum officinarum*, zma: *Zea mays*. Underlined accession numbers of genomic sequences indicate an identity > 95% to the EST sequence. n.e.: not examined, n.f.: not found. P: expressed in protonema tissue, G: expressed in gametophore tissue.

**Table 2:** List of computationally predicted *Physcomitrella *miRNAs using the micoHarvester program.

Name	Sequence 5'→3'	Length (nt)	Homologs	Precursor genomic	Precursor EST	Expression verified
miR156^a^	UGACAGAAGAGAGUGAGCAC	20	ath, gma, mtr, osa, ptc, ppt, sbi, sof, zma	1 (gnl|ti|850661024)	n.f.	n.e.
miR160-1*	UGCCUGGCUCCCUGUAUGCCA	21	ath, gma, mtr, osa, ptc, zma, sbi	1 (gnl|ti|1003375177)	n.f.	n.e.
miR160-2	CGCCUGGCUCCCUGUAUGCCA	21	ath, gma, mtr, osa, ptc, zma, sbi	1 (gnl|ti|893498247)	n.f.	n.e.
miR160-3	CGCCUGGCUCCCUGCAUGCCA	21	ath, gma, mtr, osa, ptc, zma, sbi	1 (gnl|ti|1023106236)	n.f.	n.e.
miR160-4	CGCCUGGCUCCCUGCAUGCCG	21	ath, gma, mtr, osa, ptc, zma, sbi	1 (gnl|ti|1003194173)	n.f.	n.e.
miR165	UCGGACCAGGCUUCAUUCCCCU	22	ath	1 (gnl|ti|1036028061)	n.f.	n.e.
miR166	UCGGACCAGGCUUCAUUCCCU	21	ath, gma, mtr, osa, ptc, zma, sbi	1 (gnl|ti|1006181867)	n.f.	n.e.
miR167	GGAAGCUGCCAGCAUGAUCCU	21	ath,gma,ptc,osa, sbi,sof,zma	1 (gnl|ti|1003199194)	n.f.	no
miR171-1*	AGAUUGAGCCGCGCCAAUAUC	21	ath, mtr, osa, ptc, sbi, zma	1 (gnl|ti|1024468070)	n.f.	n.e.
miR171-2	UUGAGCCGGGCCAAUAUCACA	21	ath, mtr, osa, ptc, sbi, zma	1 (gnl|ti|998754788)	n.f.	n.e.
miR172	AGAGAUUCUUGAUGAUGCUGAC	22	ath, gma, osa, ptc, sbi, zma	n.f.	1 (PR_miR172)	no
miR319-1	UUGGACUGAAGGGAGCUCCA	20	ath, gma, mtr, ptc, ppt	1 (gnl|ti|862775458)	n.f.	n.e.
miR319-2	CUCGGACUGAAGGGAGCUCCC	21	ath, gma, mtr, ptc, ppt	1 (gnl|ti|997238281)	n.f.	n.e.
miR390-1	GAGCUCAGGAGGGAUAGCGCC	21	ath, ptc, ppt, osa	n.f.	1 (PR_miR390-1)	n.e.
miR390-2^a^	AAGCUCAGGAGGGAUAGCGCC	21	ath, ptc, ppt, osa	2 (gnl|ti|866247913, gnl|ti|830400956)	n.f.	n.e.
miR395	CUGAAGCGUUUGGGGGAAAGG	21	ath,mtr,osa,ptc, sbi,zma	1 (gnl|ti|997006956)	n.f.	Yes (G)
miR408	CUGCACUGCAUCUUCCCUGUGC	22	ath, osa, ptc, sof, zma	n.f.	1 (PR_miR408)	Yes (G)
miR414	UCAUCCUCAUCAUCCUCGUCC	21	ath, osa	1 (gnl|ti|759459888)	n.f.	Yes (P)
miR418	ACAUGUGAUGAAGAACUGACA	21	ath, osa	n.f.	1 (PR_miR418)	no
miR419	UGAUGAAUGAUGACGAUGUAU	21	ath, osa	n.f.	1 (PR_miR419)	Yes (P/G)
miR473-1	CCUCUCCCUCAAAGGCUUCCA	21	ptc	n.f.	1 (PR_miR473-1)	n.e.
miR473-2	CCUCUCCCUCAAGGCUUCCA	20	ptc	1 (gnl|ti|1042068147)	n.f.	yes (P/G)
miR477	UUCUCCCUCAAAGGCUUCCAA	21	ptc	n.f.	2 (PR1_miR477, PR2_miR477)	yes (P/G)
miR533-1^a^	GAGCUGGCCAGGCUGUGAGGG	21	ppt	1 (gnl|ti|1006116182)	1 (PR_miR533-1)	n.e.
miR533-2	GAGCUGUCCAGGCUGUGAGGG	21	ppt	1 (gnl|ti|1017424894)	n.f.	n.e.
miR534-1^a^	UAUGUCCAUUGCAGUUGCAUAC	22	ppt	1 (gnl|ti|890445342)	1 (PR_miR534-1)	n.e.
miR534-2	UAUGUCCAUUACAGUUGCAUAC	22	ppt	1 (gnl|ti|1029229383)	n.f.	n.e.
miR535-1^a^	UGACAACGAGAGAGAGCACGC	21	osa, ppt	1 (gnl|ti|1020618162)	n.f.	n.e.
miR535-2	UGACAUCGAGAGAGAGCACGC	21	osa,ppt	1 (gnl|ti|1005915069)	n.f.	n.e.

^a ^Identified previously in *Physcomitrella *[17, 46]. * Identical to a miRNA in other plant species. ath: *Arabidopsis thaliana*, gma: *Glycine max*, mtr: *Medicago truncatula*, osa: *Oryza stiva*, ptc: *Populus trichocarpa*, ppt: *Physcomitrella patens*, sbi: *Sorghum bicolor*, sof: *Saccharum officinarum*, zma: *Zea mays*. Underlined accession numbers of genomic sequences indicate an identity greater than 95% to the EST sequence. n.e.: not examined, n.f.: not found. Different miRNA families are separated by lines. P: expressed in protonema tissue, G: expresses in gametophore tissue.

**Table 3:** Characterization of predicted *Physcomitrella *miRNA targets. Sequences of predicted *Physcomitrella *targets can be found at *Physcomitrella patens *resource cosmoss [77].

miRNA	Predicted *Physcomitrella *target	Gene annotation (Best hit identified by BLASTX)	Category	Comment	Hits significance
1–22	T_1-22	MUR3; Xyloglucan galactosyltransferase [*Arabidopsis thaliana*]	Cell wall		1,00E-40
1–39	T_1-39	Mucin-like protein [*Oryza sativa *(japonica cultivar-group)]	Cell wall	Water-holding	5,00E-36
1–63	T1_1-63	Putative protein kinase (Dsk1) *[Arabidopsis thaliana*]	Regulation		6,00E-70
	T2_1-63	OJ000315_02.1; similar to protein phosphatase type 2C [*Oryza sativa *(japonica cultivar-group)]	Regulation		2,00E-16
	T3_1-63	Peptidyl-prolyl cis-trans isomerase, cyclophilin type [*Medicago truncatula*]	Regulation/Defense	Cyclophilins are predicted targets in mammals	2,00E-11
2–28	T_2-28	No significant hit found.			
2–42	T_2-42	Rhodanese like protein [*Arabidopsis thaliana*]	Defense/S-Metabolism	Detoxification; in *Arabidopsis *miR396b is predicted to target a rhodanese-like domain containing protein [78]	1,00E-50
2–88	T1_2-88	Phosphoglycerate dehydrogenase-like protein [*Arabidopsis thaliana*]	N-Metabolism	Serine metabolism; only needed in non-photosynthetic organs	0.0
	T2_2-88	No significant hit found.			
3–14	T1_3-14	Membrane protein-like [*Oryza sativa *(japonica cultivar-group)]	n.a.		3,00E-19
	T2_3-14	No significant hit found.			
3–36	T_3-36	No significant hit found.			
3–79	T_3-79	Ferredoxin-nitrite reductase [*Physcomitrella patens*]	N-Metabolism		4,00E-168
3–91	T1_3-91	Kelch repeat-containing F-box family protein, putative, expressed [*Oryza sativa *(japonica cultivar-group)]	Regulation	F-Box proteins are predicted targets of miR393 and miR394 in Arabidopsis [78]	7,00E-57
	T2_3-91	No significant hit found.			
4–67	T_4-67	ThiJ/PfpI [*Mycobacterium *sp. KMS]	S-Metabolism	Thiamin biosynthesis	5,00E-36
5–21	T_5-21	Basic 2S albumin [*Helianthus annuus*]	Seed/Spore	Seed storage protein	6,00E-22
5–33	T_5-33	Conserved hypothetical protein [*Medicago truncatula*]	n.a.		7,00E-21
miR160-1, miR160-2	T_miR160-1/2	Aspartate aminotransferase (EC 2.6.1.1) [*Pinus pinaster*]	N-Metabolism	Several isoenzymes known from *Arabidopsis*, differential accumulation of mRNAS w.r.t. organ	3,00E-38
miR160-1, miR160-2, miR160-3, miR160-4	T_miR160-1/2/3/4	Auxin response factor 10 [*Oryza sativa*]	Regulation	Transcription factor	7,00E-131
miR160-2, miR160-3, miR160-4	T_miR160-2/3/4	No significant hit found.			
miR160-3	T_miR160-3	Intracellular pathogenesis-related protein-like protein [*Physcomitrella patens*]	Defense		7,00E-68
miR166	T_miR166	Class III homeodomain-leucine zipper protein HB10 [*Physcomitrella patens*]	Regulation	Transcription factor	0.0
miR167	T1_miR167	Putative mitochondrial processing peptidase [*Oryza sativa*]	Protein localisation	Cleaves signal peptide in mitochondria	3,00E-148
	T2_miR167	Delta-COP (coatomer delta subunit) [*Zea mays*]	Protein localisation	Vesicle transport, poplar miR168 is predicted to target vesicle coat protein complex COPI [79]	2,00E-63
miR171-1	T_miR171-1	UDP-N-acetylglucosamine pyrophosphorylase-like [*Oryza sativa*]	Cell wall		6,00E-14
miR319-1	T_miR319-1	Nucleotide binding [*Arabidopsis thaliana*] & WD repeat protein-like [*Arabidopsis thaliana*]	Regulation	Putative transcription factor	2,00E-109 & 4,00E-93
miR408	T1_miR408	Protein carrier [*Arabidopsis thaliana*]	Protein localisation	Predicted targets for miR408: Peptide chain release factor; plantacyanin [59]	2,00E-39
	T2_miR408	Copper ion binding/electron transporter [*Arabidopsis thaliana*] gb|AAB95306.1| putative phytocyanin [*Arabidopsis thaliana*]	Defense	Redox catalyst	2,00E-16
	T3_miR408	Phytocyanin homolog [*Pinus taeda*]	Defense	Redox catalyst	1,00E-14
	T4_miR408	Hypothetical protein P0592C05.16 [*Oryza sativa*]	n.a.		9,00E-11
	T5_miR408	Putative blue copper binding protein [*Oryza sativa*]	Defense	Redox catalyst	1,00E-11
miR414	T1_miR414	TIF3H1; translation initiation factor [*Arabidopsis thaliana*]	Regulation		4,00E-131
	T2_miR414	Hypothetical protein OSJNBb0016H12.28 [*Oryza sativa*]	n.a.		3,00E-85
	T3_miR414	Protein disulfide isomerase-like PDI-M [*Physcomitrella patens*]	Regulation	PDI is a regulator of chloroplast translational activation [80]	4,00E-67
	T4_miR414	Putative heterotrimeric G protein beta subunit [*Physcomitrella patens*]	Regulation	Signal transduction	2,00E-66
	T5_miR414	RNA binding [Arabidopsis thaliana] & Pre-mRNA splicing factor cwc22, putative, expressed [*Oryza sativa *(japonica cultivar-group)]	Regulation	Splicing/Cell cycle	2,00E-58 & 4,00E-56
	T6_miR414	AC069474_30 nascent polypeptide associated complex (NAC) alpha chain, putative [*Arabidopsis thaliana*]	Regulation	Translation, protein targeting	3,00E-40
	T7_miR414	Hypothetical protein P0665A11.10 [*Oryza sativa*]	n.a.		3,00E-25
	T8_miR414	ELM2; AT-rich interaction region; Homeodomain-related [*Medicago truncatula*] & putative MYB family transcription factor [*Arabidopsis thaliana*]	Regulation	Transcription factor (MYB)	3,00E-24 & 5,00E-23
	T9_miR414	No significant hit found.			
	T10_miR414	No significant hit found.			
	T11_miR414	No significant hit found.			
	T12_miR414	No significant hit found.			
miR418	T_miR418	Peptidyl-prolyl cis-trans isomerase, cyclophilin type [*Medicago truncatula*]	Regulation/Defense	Cyclophilins are predicted targets in mammals	1,00E-91
miR419	T_miR419	Dreg-2 like protein [*Oryza sativa*] & phospho-glycolate phosphatase [*Arabidopsis thaliana*]	Regulation	Signal transduction phosphatase	8,00E-82 & 2,00E-79
miR473-2	T1_miR473-2	Protein translocase/protein transporter [*Arabidopsis thaliana*]	Protein localisation		8,00E-38
	T2_miR473-2	PSAG_ARATH Photosystem I reaction center subunit V, chloroplast precursor (PSI-G) [*Arabidopsis thaliana*]	Photo-synthesis		2,00E-32
	T3_miR473-2	No significant hit found.			
miR477	T1_miR477	Peptidylprolyl isomerase D (cyclophilin D) [*Rattus norvegicus*]	Regulation/Defense	Cyclophilins are predicted targets in mammals	6,00E-43
	T2_miR477	Transcription factor/zinc ion binding CONSTANS-like [*Arabidopsis thaliana*]	Regulation	Transcription Factor	1,00E-22
	T3_miR477	Transcription factor/zinc ion binding CONSTANS-like [Arabidopsis thaliana]	Regulation	Transcription factor	2,00E-20
	T4_miR477	Putative cyclophilin-40 [*Oryza sativa*]	Regulation/Defense	Cyclophilins are predicted targets in mammals	4,00E-16
	T5_miR477	No significant hit found.			
	T6_miR477	No significant hit found.			
miR473-1, miR477	T_miR473-1/miR477	No significant hit found.			
miR533-2	T_miR533-2	Hydrolase-like protein [*Oryza sativa*] & chloroplast Phyllo [*Arabidopsis thaliana*]	Metabolism/Regulation	Phylloquinone biosynthesis; homology to lysophospholipase region of PHYLLO locus.	5,00E-16 & 2,00E-14
miR534-1	T1_miR534-1	Inorganic pyrophosphatase [*Nitrosospira multiformis*]	Metabolism		4,00E-21
	T2_miR534-1	BTB/POZ domain protein (BLADE-ON-PETIOLE 2) [*Arabidopsis thaliana*]	Regulation	Leaf and floral patterning	7,00E-11

Experimentally verified targets are underlined, n.a.: no category assignment.
