# Supplementary material for: Evidence for the rapid expansion of microRNA-mediated regulation in early land plant evolution
Source: BMC Plant Biol. 2007 Mar 14;7:13. doi: 10.1186/1471-2229-7-13 (PMC1838911; doi:10.1186/1471-2229-7-13)
Supplement: Additional file 1 — Non redundant set of cloned sRNA sequences after filtering. The table shows the complete non-redundant set of sRNA sequences which have been obtained from the sRNA cloning approach. Only those sRNA sequences are listed which did not show homologies to rRNA, tRNA or chloroplast encoded RNA sequences deposited in Rfam. [file 1471-2229-7-13-S1.pdf]

Additional file 1 Non redundant set of cloned sRNA sequences after filtering.

Clone ID	Sequence 5'→3'	Length (nt)	Frequency	Hit in the genomic trace files and/or EST database
1-1	AGGCGACGGGGAUUCA	16	1	
1-46	GGCUACUUCGGCGAAA	16	1	
1-7	GAGCUUUCUUCGGUCC	16	1	
2-8	UGCGGAAGGAUCAUUG	16	1	
3-15	AUCCGAUGAGGGUGUA	16	1	
4-75	GUGACGCGCAUGAAUG	16	1	
2-67	AGACCCAGUACUACAGC	17	1	
3-13	AGCGUCGAGUGCAGAUC	17	1	
3-21	CUGCUGAGCACGCCAAA	17	1	
3-61	CCAUAUCGUUCUUUAAA	17	1	
4-53	GAGGUCUGGGGGGUGAG	17	1	
5-40	AGGCUAGCCAGGCGGUG	17	1	
1-15	UCAUGCCCGCUCGUACAA	18	1	
1-16	CUCUCAGUGGAAGCACCA	18	1	
1-74	CUGCUAAUACAGCUCCAC	18	1	
2-2	GCCCGCGCAGGUUCGAAA	18	1	
2-61	GCCCCCAUCGUCUAGUGA	18	1	
2-66	CCUGCGGAAGGAUCAUUG	18	1	
3-1	GCGGGGAUAGCUCAGUUG	18	2	
3-46	CAACAGCUAUUCCAUCUA	18	1	
4-55	ACUAUGCCUGAGCAGGGA	18	1	
5-11	GCAUGAACGUUUCCAAAC	18	1	
5-54	GUGACGCGCAUGAAUGGA	18	1	
2-23	AUAGACAAAAGUGGUAUAU	19	1	
2-70	GUUGGAAGCCUUCGUGGGA	19	2	yes
3-72	UUGGUACACUUGACGCAAA	19	1	
4-14	CCCGCCCACCCUGGAAUCG	19	1	
4-31	GGCGGACGUGGCCAAGUGG	19	1	
4-64	GUUGCUUGGUUGAACCCCG	19	1	
5-41	AGUUGGAUCUUGGGUAAAA	19	1	
1-22	AUUGGGACUUGUGCUGGGAC	20	1	yes
1-31	CGGAGGUCUGCCCGAGCUGC	20	1	yes
1-39	CGUUUCACGUCGGGUUCACC	20	1	yes
1-41	GCCGGGAUAGCUCAGUUGGA	20	1	
1-63	UUGCUGUGCACUACUUAGUA	20	1	yes
2-51	GAGCUUUCUUCGGUCCAAUA	20	1	yes
2-88	UGACAGAAGAGAGUGAGCAC	20	1	yes
2-90	GGCAUUCCUGCCAGAGCGUC	20	1	yes
2-93	GCGGGGAUAGCUCAGUAAAA	20	1	
2-95	UAGGGACGGUUGGGGGCAAA	20	1	

3-29	ACCAAUACCAGGUCCAAUAG	20	1	yes
3-42	AGGAGGGCGUGGGGGUCGUG	20	1	yes
3-51	CAUCAUGUUAGUCAGCCUGG	20	1	yes
3-6	UUGGAAGAGCAGAUCGAAUU	20	1	
3-91	GCUGUGUUCUUGUACCUGGG	20	1	yes
3-96	UACCUGGUUGAUCCUGCCAG	20	1	yes
4-28	CCUGCGGAAGGAUCAUUGAA	20	1	yes
4-33	CUGAAGACCGCCUUUACAAA	20	1	
4-65	GCGCUUUUAGUUCAGUCUGG	20	1	yes
4-68	GUCGAUAUGGCCGAGAAAAA	20	1	
5-21	UCUUGUCAAUGUUUAGGGGC	20	1	yes
5-28	AACGUGAUAGGACUCUGAAA	20	1	
5-33	UUGAGGUGUUUCUACAGGCU	20	1	yes
5-51	CGAACAGCCGGUUUUCAAGU	20	1	yes
5-70	UGCCGAAGCUGUGGGAUGUC	20	1	yes
1-36	GUUGGGCCUAGGGGUCUGUGA	21	1	yes
1-50	UGGCUGAGUCGAAGGUUGUGC	21	7	yes
1-67	UCCAGGCCCGGACAACGUAAC	21	1	
2-28	CGCUGUCCAUUCUGAGCAUUG	21	1	yes
2-31	UGACAACGAGAGAGAGCACGC	21	9	yes
2-33	UCGAGUCCGCUUGUCUCCACC	21	1	yes
2-38	ACAGACCGAGAGAUUUGAACA	21	1	yes
2-44	CAAGUCCAGGAUAGCCCACCA	21	1	yes
2-86	CCUUAGAGUCGUAGGCCUCUG	21	1	yes
2-92	ACUAAGGCAUGCCAAACAUUA	21	1	yes
3-14	GCUAGGCAGUGCACAGCGAUA	21	4	yes
3-49	CCCUCGUCGGCCCUUGAAAAA	21	1	
3-62	AACUGAGAUACAUCGCAAUCG	21	1	yes
3-71	CCUUAGGCUCGGAGGUCUGCA	21	1	
3-79	UCAUCCAGGGAGCCAGACAGA	21	1	
4-12	GGUAAAGUGGCGGCUAGGUUA	21	1	yes
4-19	CCAUGGCCGACUCUGAGCAGC	21	1	yes
4-27	CUGCGGAAGGAUCAUUGAAAA	21	1	yes
4-29	GGAGAGAUGGCCGAGUGGCUU	21	1	yes
4-34	CGUGGGACAGCAUAGAAUGCG	21	1	yes
4-45	CGGAGGUCUGCCCGAGCUGCU	21	1	yes
4-72	UUGAGCCGCGCCAAUAUCACA	21	1	yes
4-9	GGUGGCUGUAGUUUAGAAAAA	21	1	
5-27	UCCUGCCAGAGCGUCAUCAAA	21	1	
5-42	UCCAUUUAGCAGCUUCACGAC	21	1	
5-50	GCUACAGCAGCUCCACCAAAA	21	1	
1-68	AGACACGACGACGGCCCAUAUC	22	1	
1-81	UGGCUGAGUCGAAGGUUGUGAA	22	1	
2-1	GUAGCUUAGCGAGGUGUUGGUA	22	1	yes

2-15	UGACAACGAGAGAGAGUACGCU	22	1	
2-4	UCGAGUCCGCUUGUCUCCACCA	22	2	yes
2-84	CAACUGCAGGUGCAGGACCAUG	22	1	
2-85	CAUCGCCGCAAUACCUAAAGUU	22	1	yes
2-96	CCCGAAGUCGUUACCCUAACAA	22	1	yes
3-22	CUGAUCAUGCCCGGUCGUACAA	22	1	yes
3-36	GCUACUUCGGCGGGACAAGAGA	22	1	yes
3-40	UGACAGAAGAGAGUGAGCACAU	22	1	
3-52	GUGGGACGUGUGGACGAAAAAA	22	1	
3-60	UUCGUGCCAAGCUGUGUGCAAC	22	5	yes
3-78	UGAAUGCAACACUCCUACGCAA	22	1	yes
4-43	GCAACCAGACUCUCUCACAAAA	22	1	
4-58	CUCACCGUCCCAACGAUUUGUA	22	1	yes
4-67	AUCGUGCCAAGCUUUGUGCUUU	22	1	yes
4-70	CUAAUGAAACUGUUAAUACACC	22	1	yes
5-23	GCAGACACGCGCGGCCCAUAUC	22	1	yes
5-48	GAACGAGACCUCAGCCUGAAAA	22	1	
5-90	GGCGGACGUGGCCAAGUGGAAA	22	1	
3-20	GUUCGAGUCCGCUUGUCUCCACC	23	4	yes
3-5	UGAUCAAGUGGAAACUCAGCAAA	23	1	yes
4-16	AACACCAACGGCGAAAGCACUAA	23	1	yes
4-66	ACGAAGGUCUGCAUCAUAGCCAA	23	1	yes
5-3	UUUUCGGUGAAAAGUGACUCUGC	23	1	yes
5-5	GUUCAAGUCCUACCUGGGGAGCC	23	2	
5-58	UUCGUUUCUCGCAGUGCCCCCCA	23	1	
5-63	AUGGUUCAAGAAGAGACACGUGG	23	1	yes
1-55	GUGGGUGACUGCCCCCCUGCCUCA	24	1	yes
2-68	CAAAAGAAUAGCUGCCCAACACAA	24	1	
3-44	UCGGAAGCCUUUGUGGGAGAGGAA	24	1	
3-54	CUUGGACUGAAGGGAGCUUUUUUU	24	1	
4-1	CUUGAUACUCUAUUGACCAUUAAC	24	1	
5-19	GAUUCGAAUUCCCCUGGGGGGACC	24	1	
5-86	GGUUCAAGUGCAGGAUAGCCCACC	24	1	
1-21	GAUUCGAAUUCCCCUGGGGGCUACC	25	1	
1-29	GACCGUGCAGCUGGAACCUUGUACA	25	1	yes
1-51	GAUUCGAAUUCCCCUGGGGGUUACC	25	1	
1-57	GUUCAAGUGCUACCUGGGGAGACCA	25	1	
2-19	GGUUCGAGUCCAGGAUAGCCCACCA	25	1	
2-42	GUCAAUUUGGCCGAGUGGUUAAGGC	25	1	yes
2-45	UUCGGAAUUUCAGUGUUCGGACAGG	25	1	yes
2-82	GGUUCAAGUCCAGGGUAGCCCACCA	25	1	
4-23	UCAUAGCCGCACCAAUGAAUAAGAA	25	1	yes
4-26	GCGUUUGUAGUCCAACGGUUAGGAU	25	1	yes
4-60	CAUUAAAGCCUUGCGAGCAGUACUA	25	1	yes

5-30	UGCUGCACCAGCUGCUGAGGAAAAG	25	1	yes
1-54	GUUCAAGUCCAGGUAUACGUCCCACC	26	1	
2-24	UGUACACACCGCCCGUCAAAAAAAAA	26	1	
2-7	AGGGAGGGAUGGUUAUGCAAAAAAAA	26	1	
3-39	AAAUUCAAAUUCCCCCUGGGGGUUAC	26	1	
3-59	UCGAAUGGGGACCUAUCGCAGAAAAA	26	1	yes
3-90	UGAGUUCAGCUCUCAGUGGAAGCACC	26	1	
4-40	CGGCGGCGACCGCAGCUAACAGGCUA	26	1	yes
4-57	CUUAUUUUUUCUAAGUGUCAUUCUA	26	1	
5-57	UGUUUCGGUGCGGGCUGCGAAAAAAA	26	1	
1-65	CAUGACUUUUGGAAUAUUGCCAUUCAG	27	1	yes
2-57	AAGAUAGAGGAGUUCAAGAAAAAAAAA	27	1	
3-47	UGCAGGCAGUGCAGCCCAAAAAAAAAA	27	1	
3-87	AAGCGGGAGGUCCUGAGUUCUCUCUCA	27	1	
4-44	UCGAAUUCCCCUGGGGGUACCAAAAAA	27	1	
4-79	CUUAUUUUUUCUAAGUGUCAUUCUAU	27	1	
5-12	CUUAUUUUUUCUAAGUGUCAUUCUAA	27	1	
5-4	GAUUACUGAUCAUGCCCGGUCGUACAA	27	1	yes
5-52	GAUUACGAAUUACACCCUGGGGGUACC	27	1	
5-72	UAAGAUUGGUGCCCCAGUAUGGAACAA	27	1	
2-14	AUCCUAACCAGCACCGAGAGAUUUGAAC	28	1	
1-23	GAUUCGAAUUACCCACUGGGGGUACCAAAA	30	1	
5-44	GAUUACGAAUUCCCCUGGGGGUACCAAGAA	30	1	
1-73	ACCUUGGCUGUCACUCAAAUCCGUUGUAGUCUAG	34	1	
