# Supplementary material for: Evidence for the rapid expansion of microRNA-mediated regulation in early land plant evolution
Source: BMC Plant Biol. 2007 Mar 14;7:13. doi: 10.1186/1471-2229-7-13 (PMC1838911; doi:10.1186/1471-2229-7-13)
Supplement: Additional file 2 — Precursor structures of Physcomitrella miRNAs. Fold back analysis of identified potential precursor sequences. Genomic sequences and EST sequences harboring regions identical to sequences of cloned and predicted sRNAs were trimmed and clustered. The non-redundant set of singlets and contigs was used for structural analysis using the RNAshapes program. The mature miRNA sequences within the precursors are highlighted in red. [file 1471-2229-7-13-S2.pdf]

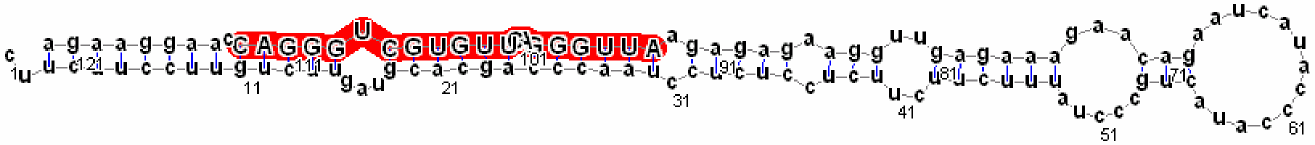

1-22 (gnl|ti|872730449)

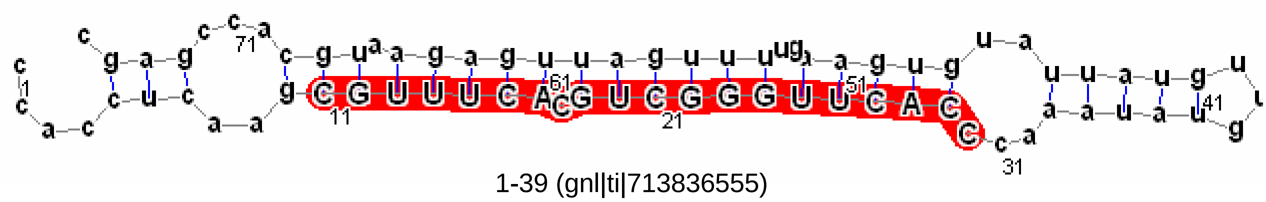

1-39 (gnl|ti|713836555)

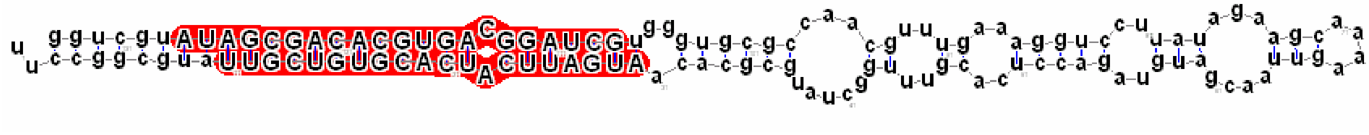

3-14 & 1-63-precursor-1 (gnl|ti|1012878547)

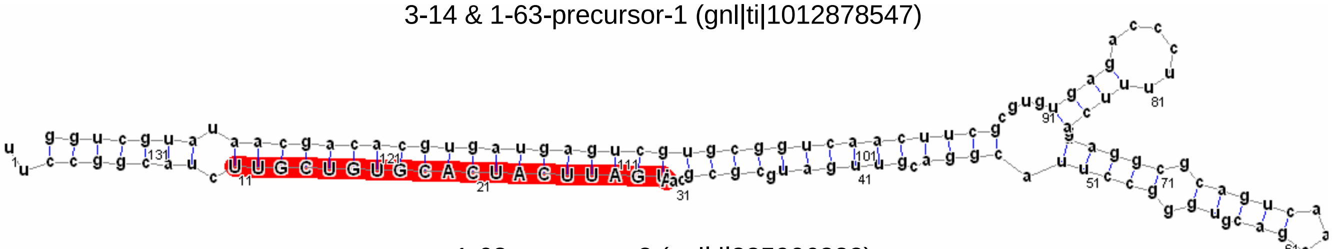

1-63-precursor-2 (gnl|ti|835906822)

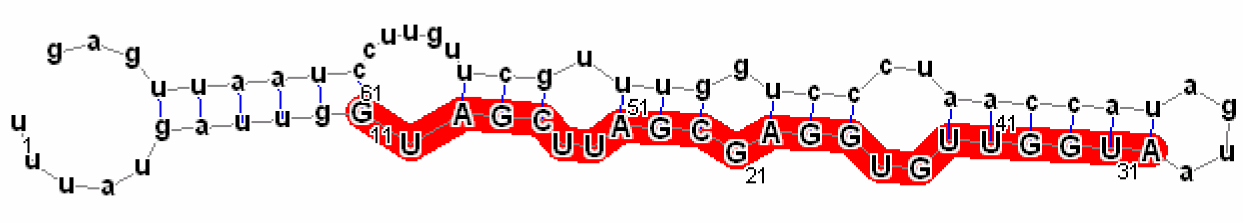

2-1 (gnl|ti|890552627)

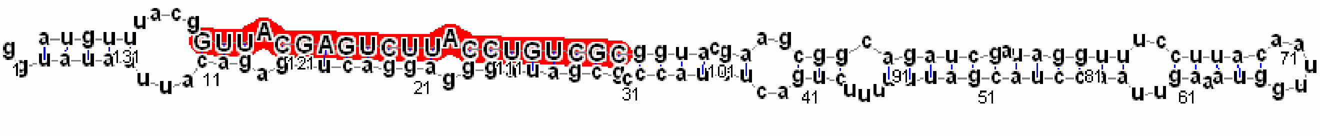

2-28 (gnl|ti|1010151671)

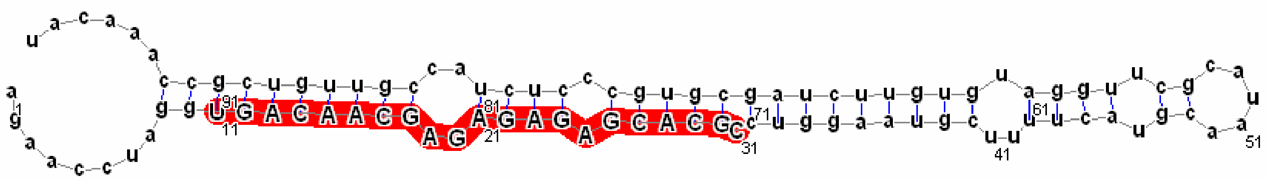

2-31-precursor1 (gnl|ti|1003237208)

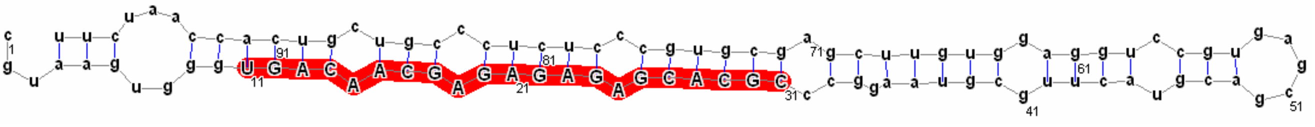

2-31-precursor2 (gnl|ti|756805268)

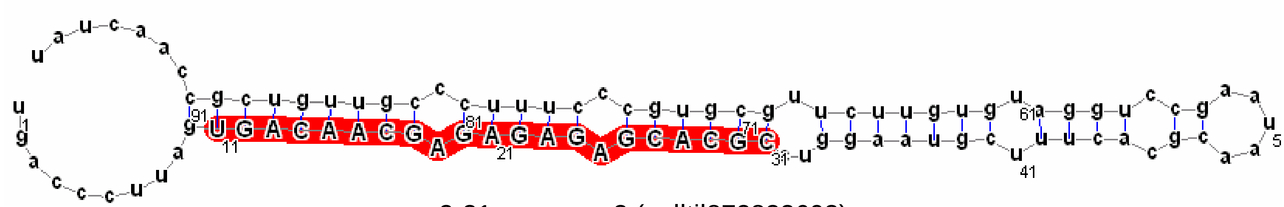

2-31-precursor3 (gnl|ti|872833603)

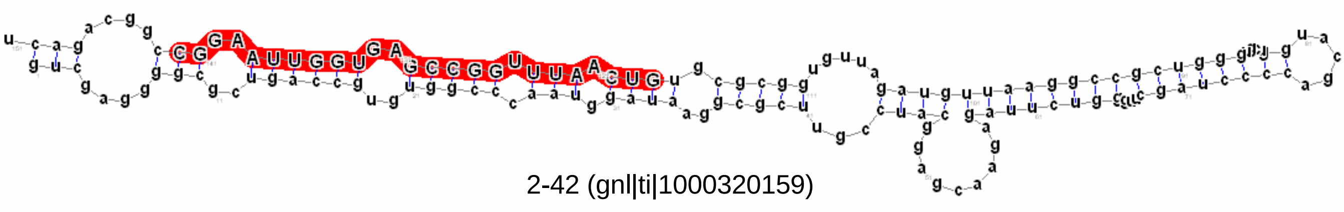

2-42 (gnl|ti|1000320159)

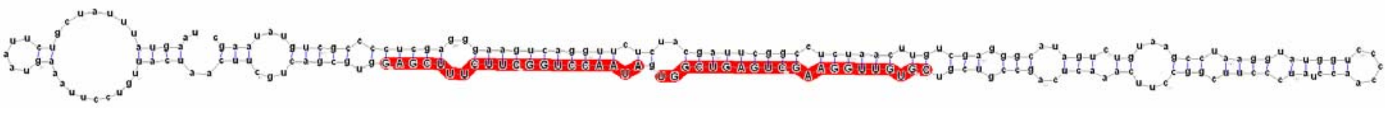

2-51 & 1-50 (gnl|ti|859644225)

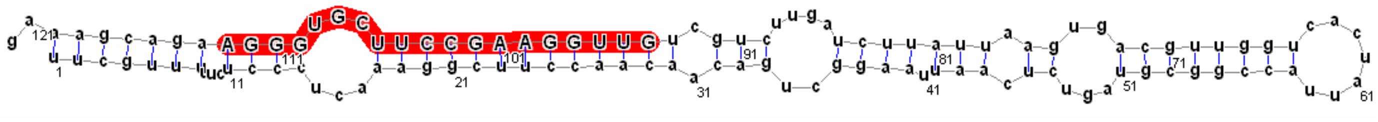

2-70 (PR_2-70)

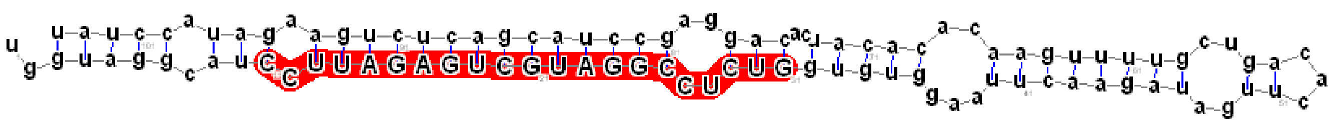

2-86 (gnl|ti|774610216)

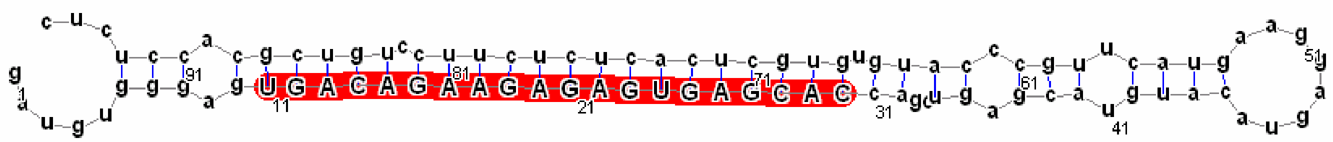

2-88-precursor1 (gnl|ti|850661024)

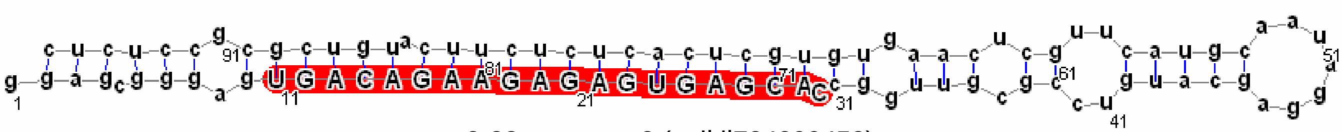

2-88-precursor2 (gnl|ti|784299453)

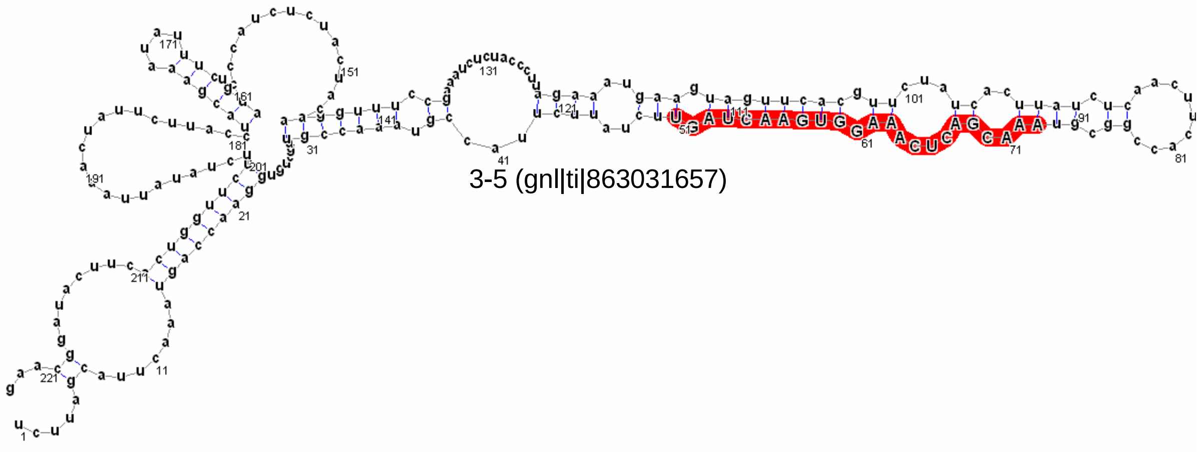

3-5 (gnl|ti|863031657)

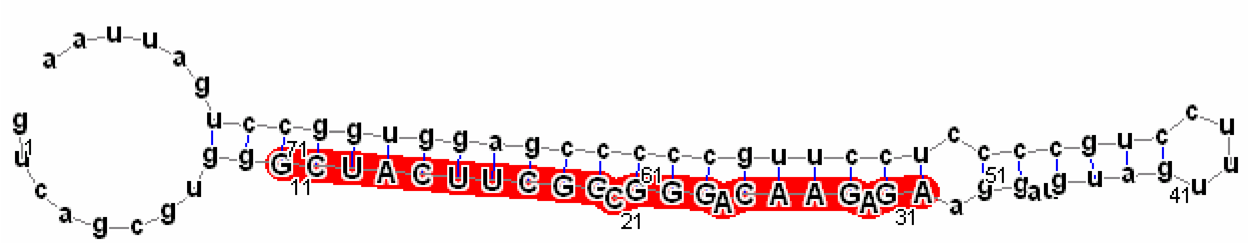

3-36 (gnl|ti|1020603193)

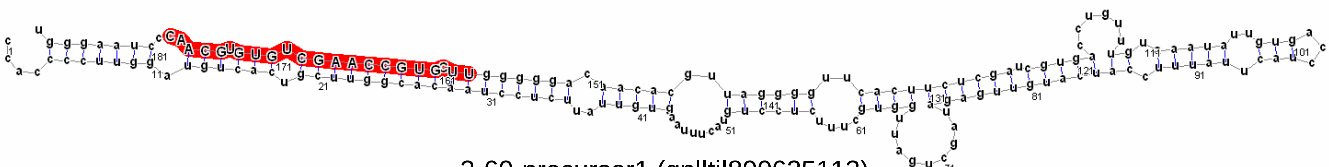

3-60-precursor1 (gnl|ti|890625113)

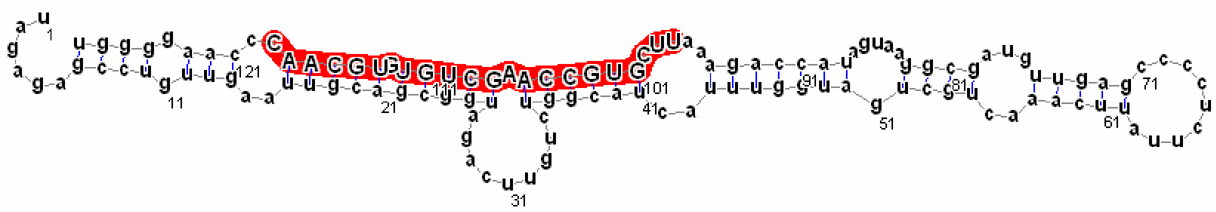

3-60-precursor2 (gnl|ti|869792930)

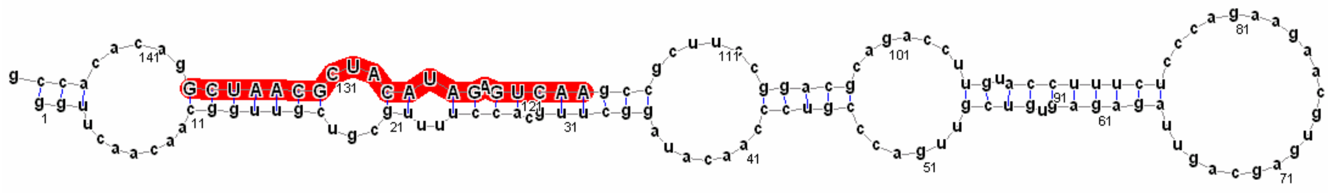

3-62 (gnl|ti|1029072876)

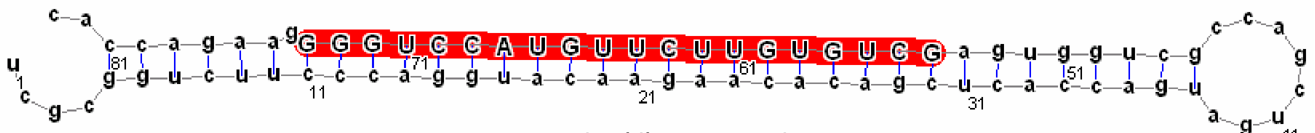

3-91 (gnl|ti|831706876)

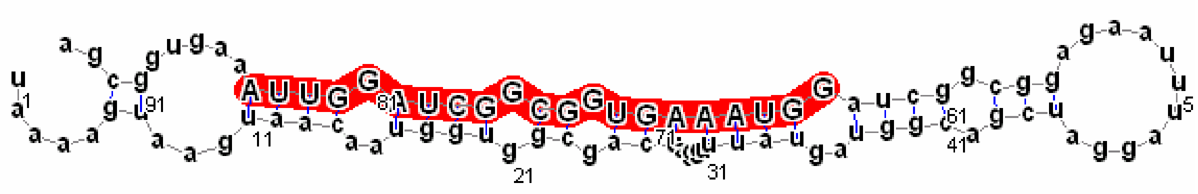

4-12 (gnl|ti|890397681)

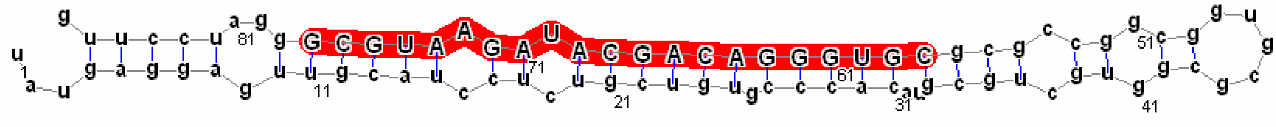

4-34 (gnl|ti|713871562)

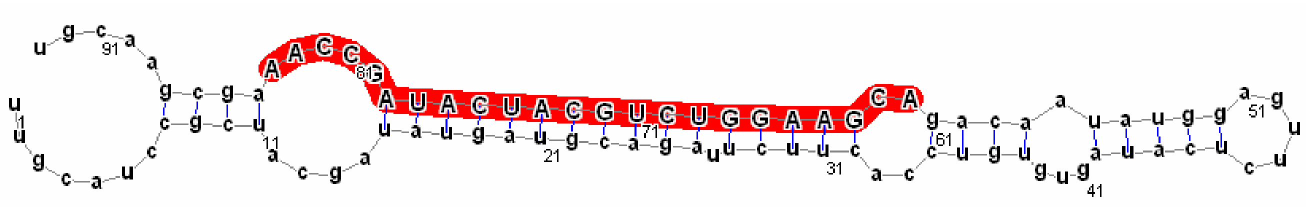

4-66-precursor1 (gnl|ti|1000325696)

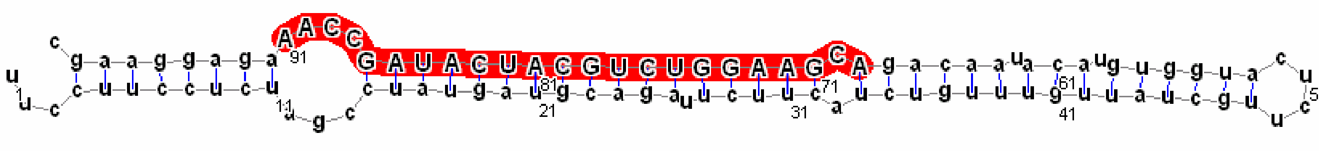

4-66-precursor2 (gnl|ti|816375179)

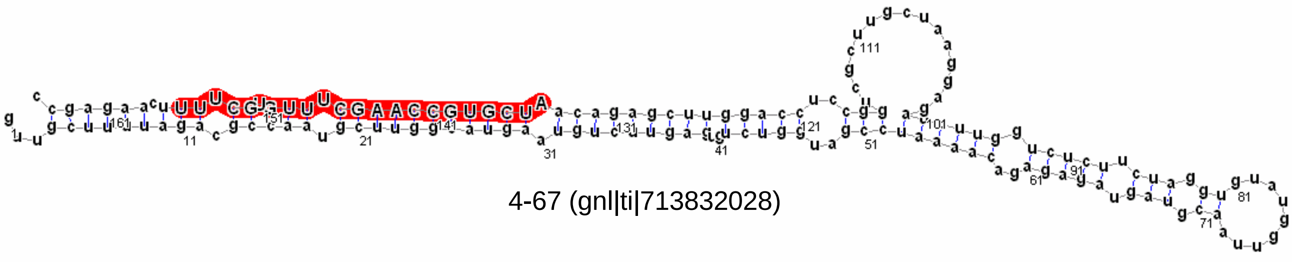

4-67 (gnl|ti|713832028)

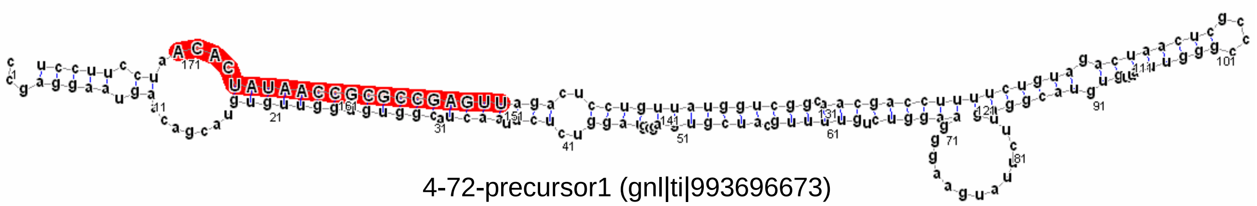

4-72-precursor1 (gnl|ti|993696673)

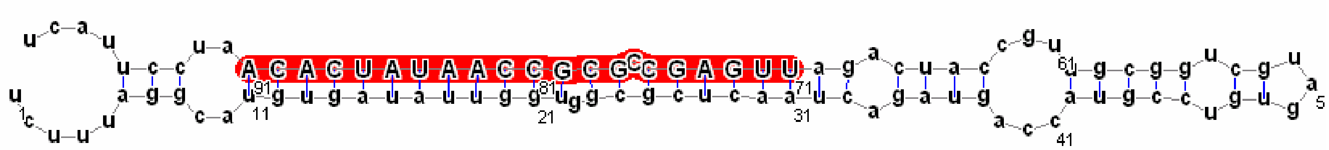

4-72-precursor2 (gnl|ti|1023219413)

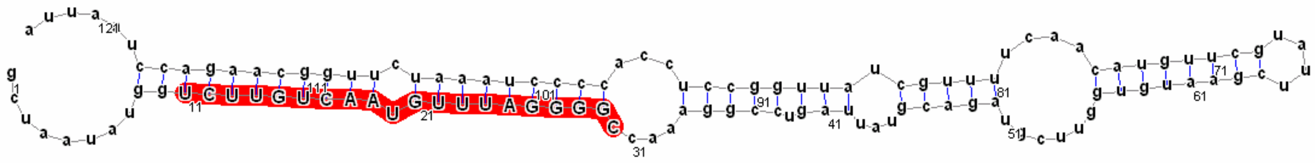

5-21-precursor1 (gnl|ti|891393071)

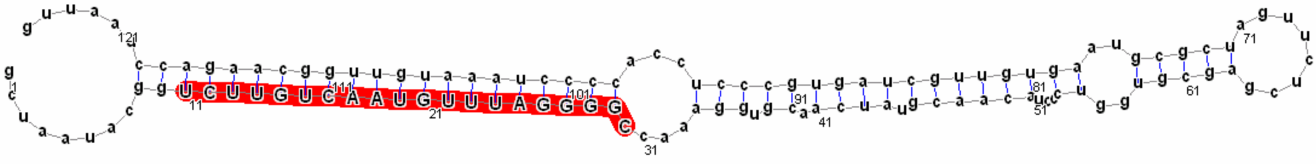

5-21-precursor2 (gnl|ti|836345675)

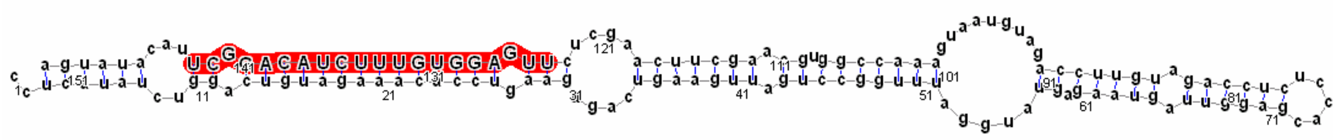
5-33 (gnl|ti|903313912)

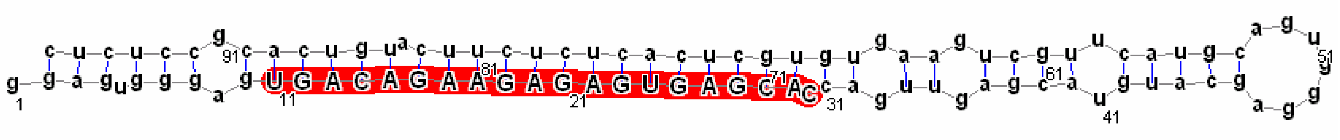
miR156 (gnl|ti|997353472)

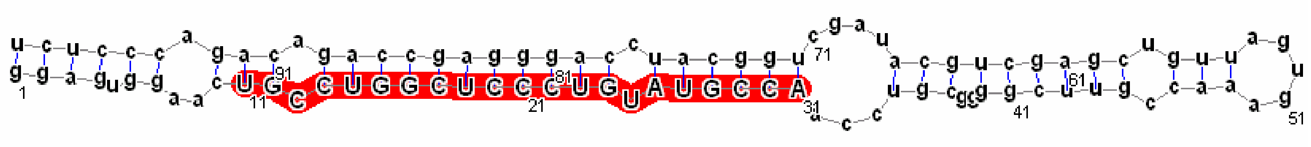
miR160-1 (gnl|ti|1003375177)

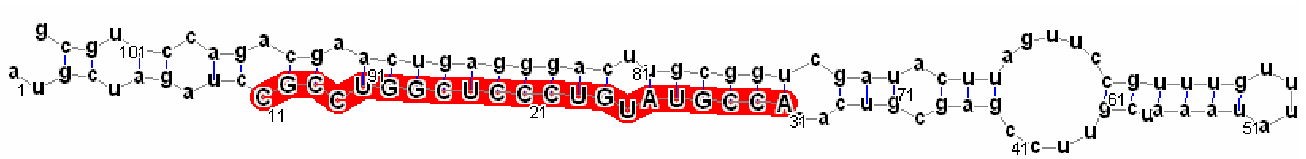
miR160-2 (gnl|ti|893498247)

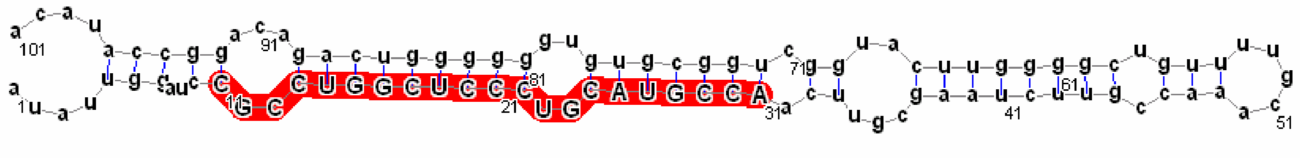
miR160-3 (gnl|ti|1023106236)

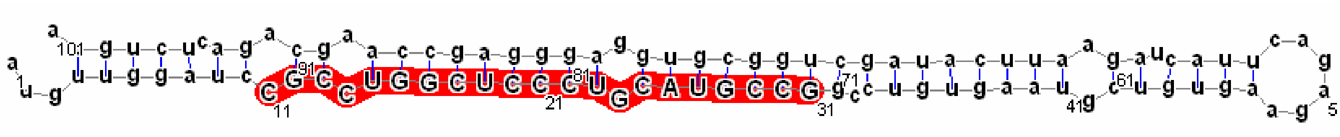
miR160-4 (gnl|ti|1003194173)

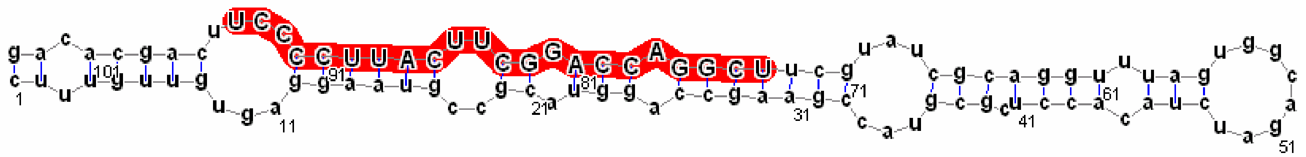
miR165 (gnl|ti|1036028061)

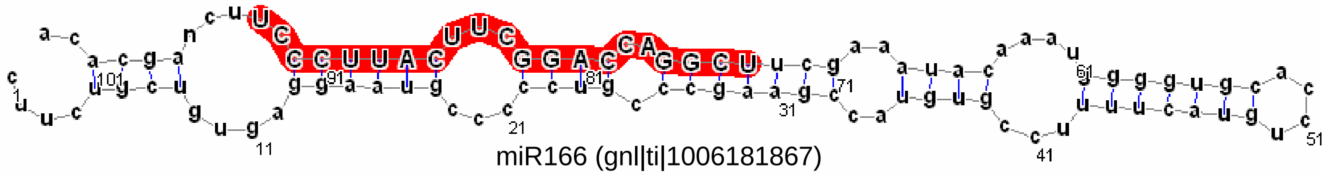
miR166 (gnl|ti|1006181867)

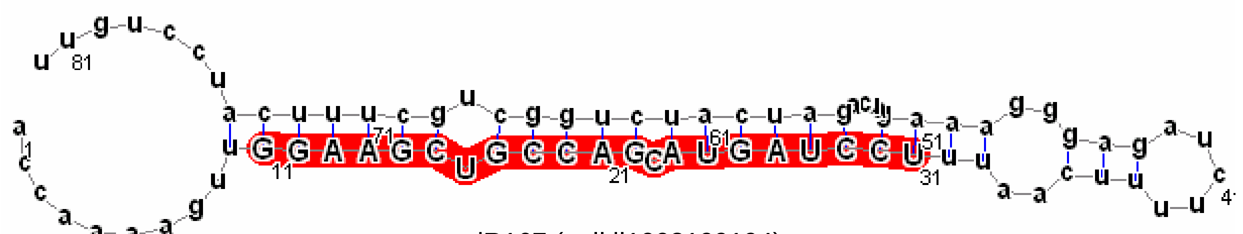
miR167 (gnl|ti|1003199194)

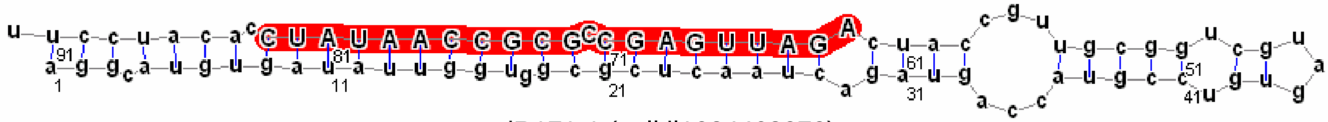

miR171-1 (gnl|ti|1024468070)

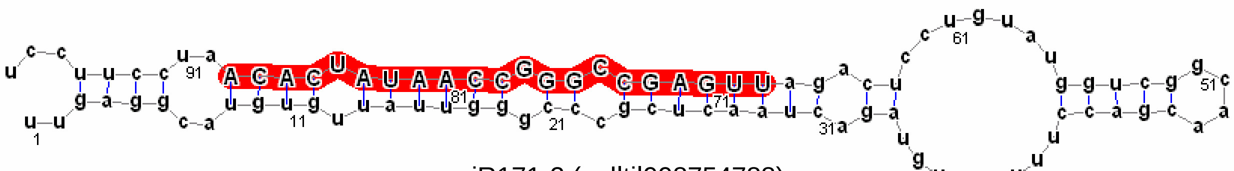

miR171-2 (gnl|ti|998754788)

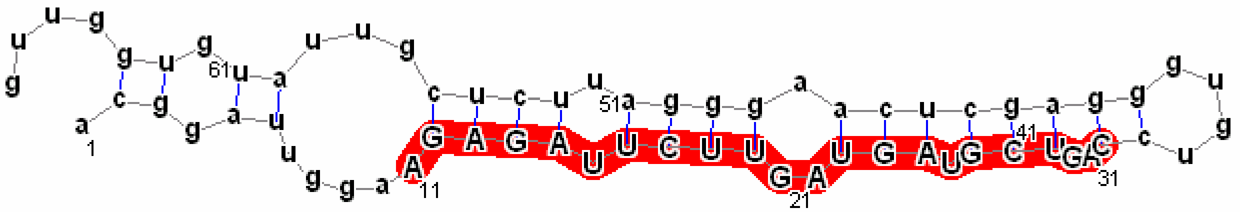

miR172 (PR_miR172)

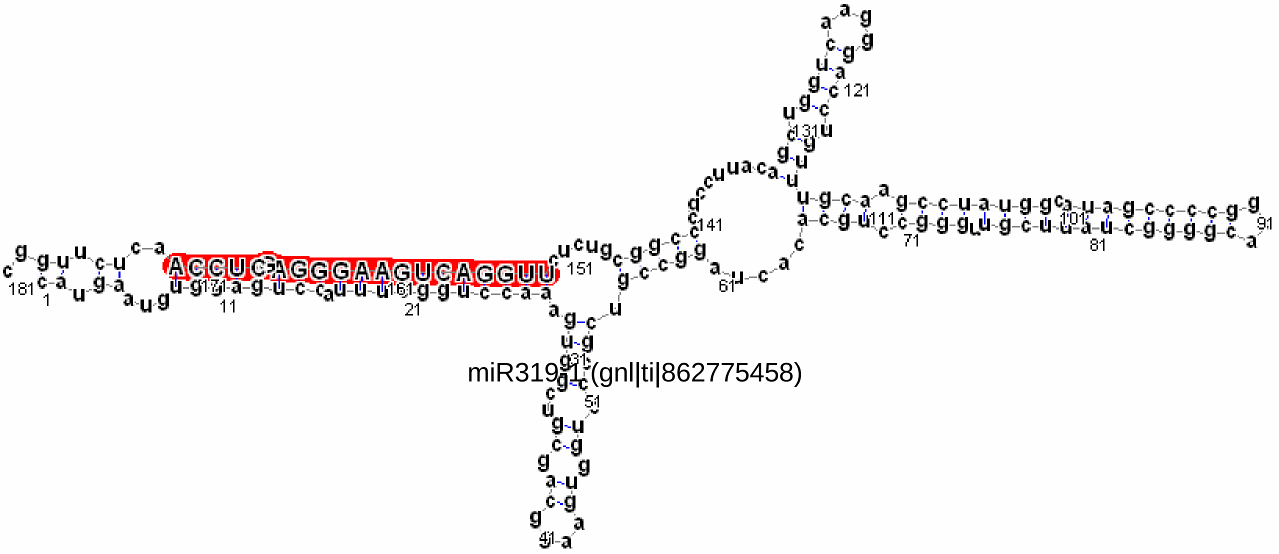

miR319-1 (gnl|ti|862775458)

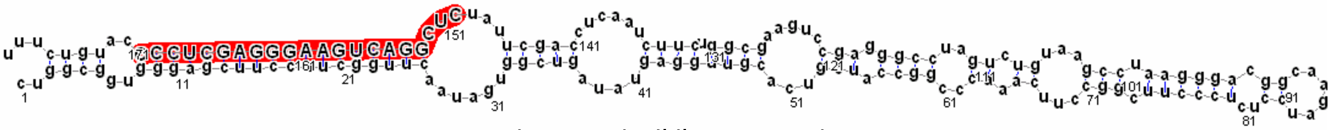

miR319-2 (gnl|ti|997238281)

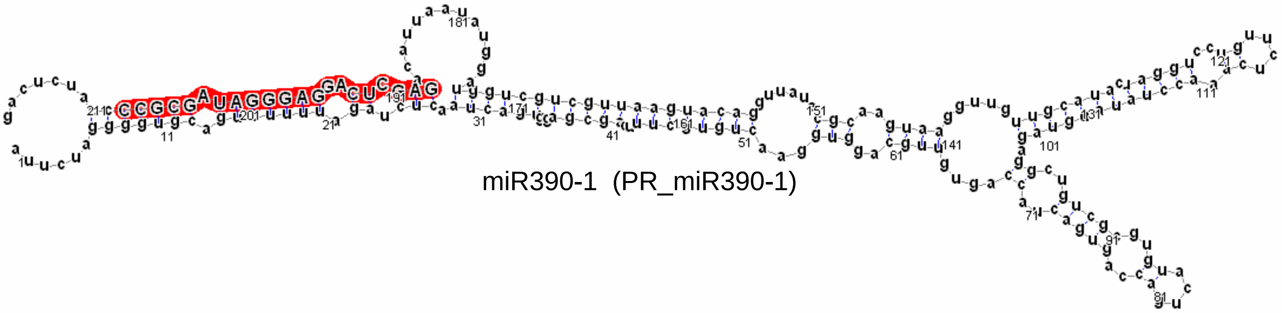

miR390-1 (PR_miR390-1)

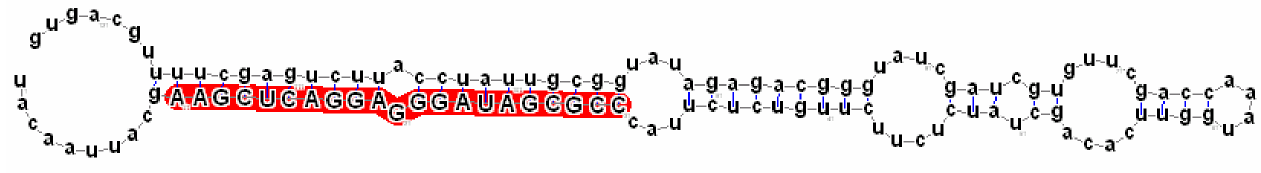

miR390-2-precursor1 (gnl|ti|866247913)

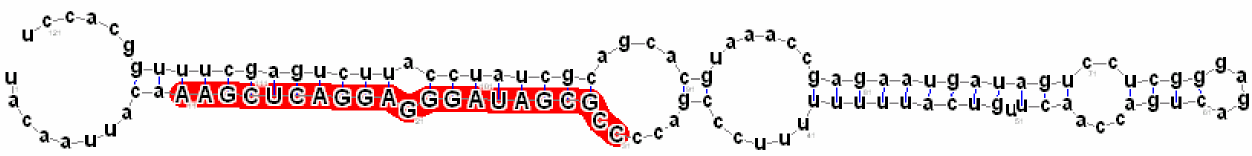

miR390-2-precursor2 (gnl|ti|830400956)

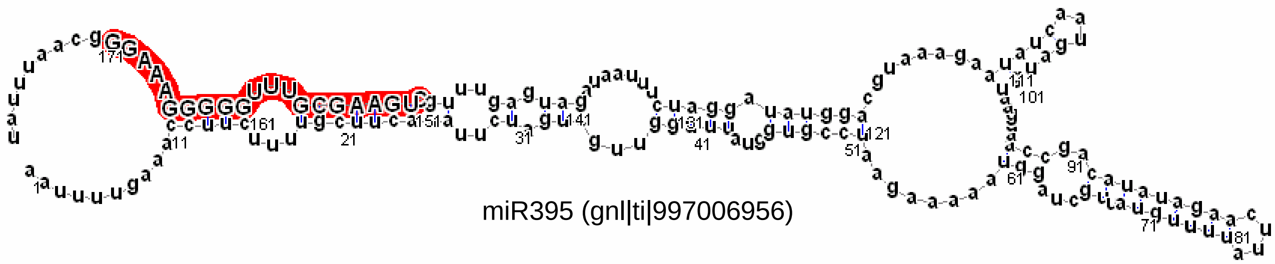

miR395 (gnl|ti|997006956)

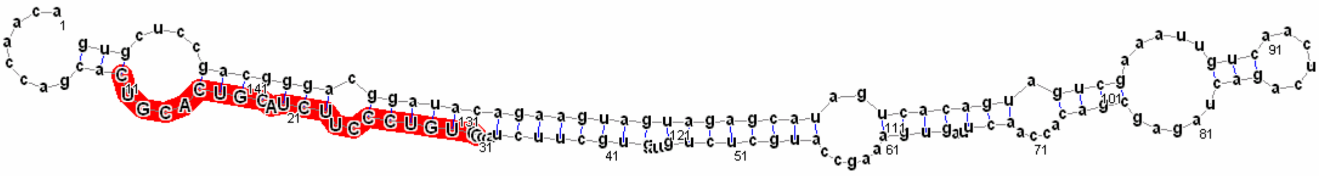

miR408 (PR_miR408)

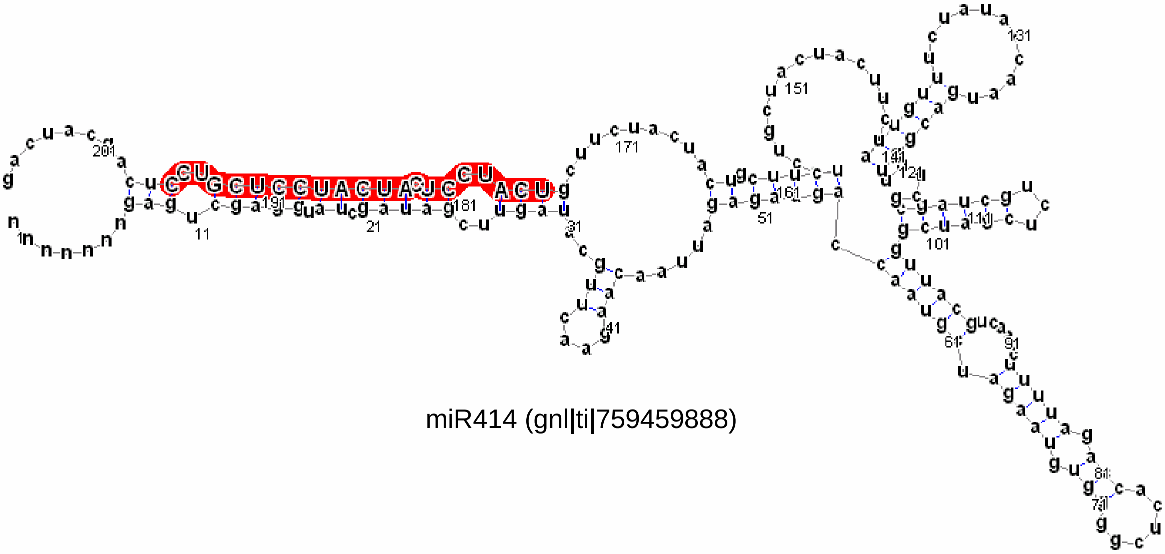

miR414 (gnl|ti|759459888)

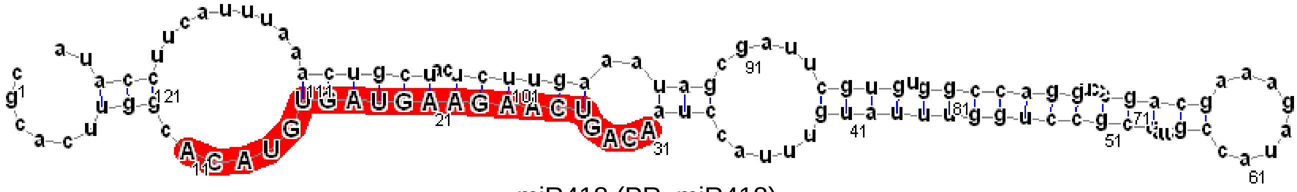

miR418 (PR_miR418)

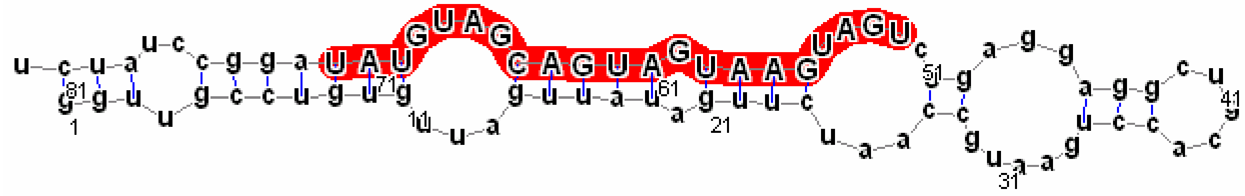

miR419 (PR_miR419)

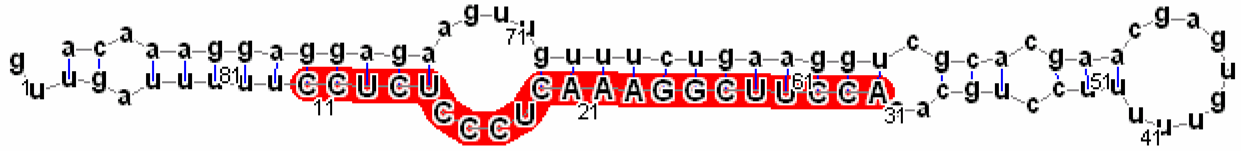

miR473-1 (PR_miR473-1)

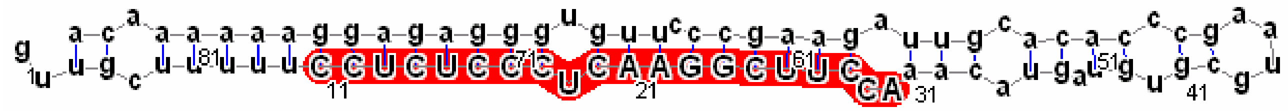

miR473-2 (gnl|ti|1042068147)

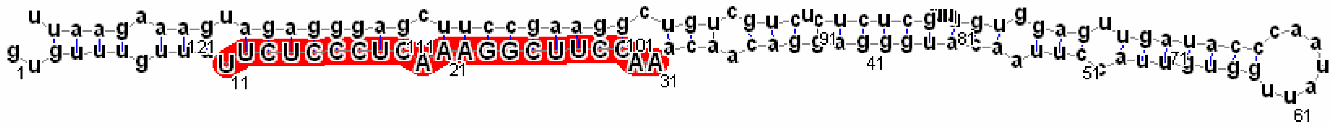

miR477-precursor1 (PR1_miR477)

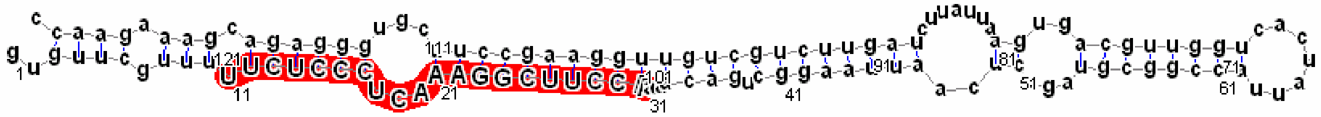

miR477-precursor2 (PR2_miR477)

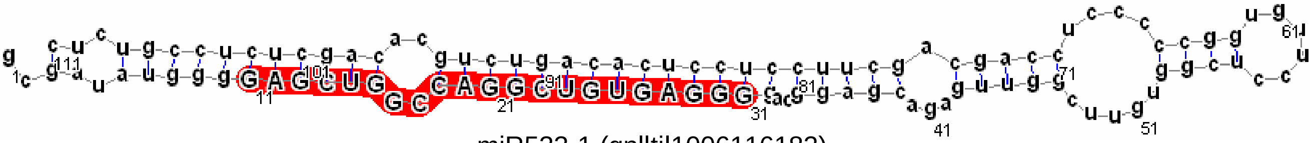

miR533-1 (gnl|ti|1006116182)

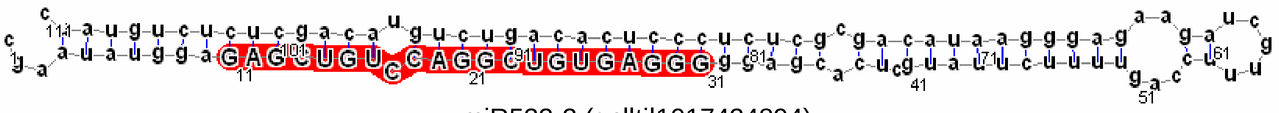

miR533-2 (gnl|ti|1017424894)

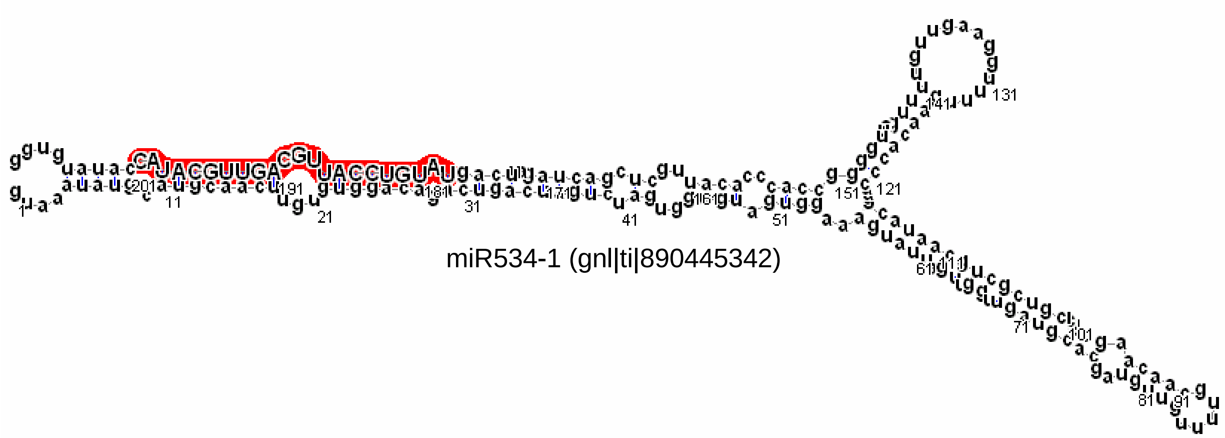

miR534-1 (gnl|ti|890445342)

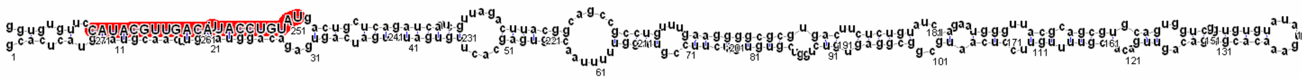

miR534-2(gnl|ti|1029229383)

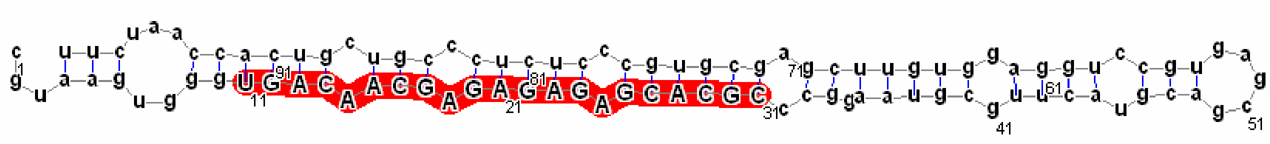

miR535-1 (gnl|ti|1020618162)

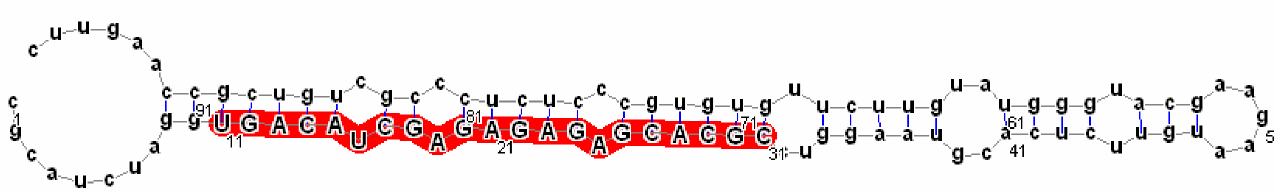

miR535-2 (gnl|ti|1005915069)
