# Supplementary material for: Evidence for the rapid expansion of microRNA-mediated regulation in early land plant evolution
Source: BMC Plant Biol. 2007 Mar 14;7:13. doi: 10.1186/1471-2229-7-13 (PMC1838911; doi:10.1186/1471-2229-7-13)
Supplement: Additional file 3 — miRBase annotations of identified Physcomitrella miRNAs. The table shows annotations of the identified Physcomitrella miRNAs which were deposited in miRBase. [file 1471-2229-7-13-S3.pdf]

Additional file 3 Annotated sequences by miRBase

Identified miRNAs	Sequence 5'→3'	miRBase annotation
1-22	AUUGGGACUUGUGCUGGGAC	ppt-miR893
1-39	CGUUUCACGUCGGGUUCACC	ppt-miR894
1-50	UGGCUGAGUCGAAGGUUGUGC	ppt-miR319d.2*
1-63 (gnl|ti|1012878547)	UUGCUGUGCACUACUUAGUA	ppt-miR898a-5p
1-63 (gnl|ti|835906822)	UUGCUGUGCACUACUUAGUA	ppt-miR898b
2-1	GUAGCUUAGCGAGGUGUUGGUA	ppt-miR895
2-28	CGCUGUCCAUUCUGAGCAUUG	ppt-miR390c*
2-31 (gnl|ti|1003237208)	UGACAACGAGAGAGAGCACGC	ppt-miR535a
2-31 (gnl|ti|756805268)	UGACAACGAGAGAGAGCACGC	ppt-miR535d
2-31 (gnl|ti|872833603)	UGACAACGAGAGAGAGCACGC	ppt-miR535e
2-42	GUCAAUUUGGCCGAGUGGUUAAGGC	ppt-miR896
2-51	GAGCUUUCUUCGGUCCAAUA	ppt-miR319d.1*
2-86	CCUUAGAGUCGUAGGCCUCUG	ppt-miR1218
2-88 (gnl|ti|850661024)	UGACAGAAGAGAGUGAGCAC	ppt-miR156a
2-88 (gnl|ti|784299453)	UGACAGAAGAGAGUGAGCAC	ppt-miR156b
3-5	UGAUCAAGUGGAAACUCAGCAAA	ppt-miR897
3-14	GCUAGGCAGUGCACAGCGAUA	ppt-miR898a-3p
3-60 (gnl|ti|890625113)	UUCGUGCCAAGCUGUGUGCAAC	ppt-miR536a
3-60 (gnl|ti|869792930)	UUCGUGCCAAGCUGUGUGCAAC	ppt-miR536b
3-62	AACUGAGAUACAUCGCAAUCG	ppt-miR899
3-91	GCUGUGUUCUUGUACCUGGG	ppt-miR900
5-21 (gnl|ti|891393071)	UCUUGUCAAUGUUUAGGGGC	ppt-miR904a
5-21 (gnl|ti|836345675)	UCUUGUCAAUGUUUAGGGGC	ppt-miR904b
5-33	UUGAGGUGUUUCUACAGGCU	ppt-miR537a
4-12	GGUAAAGUGGCGGCUAGGUUA	ppt-miR901
4-34	CGUGGGACAGCAUAGAAUGCG	ppt-miR1212
4-66 (gnl|ti|1000325696)	ACGAAGGUCUGCAUCAUAGCCAA	ppt-miR902a
4-66 (gnl|ti|816375179)	ACGAAGGUCUGCAUCAUAGCCAA	ppt-miR902b
4-67	AUCGUGCCAAGCUUUGUGCUUU	ppt-miR536c
4-72 (gnl|ti|1023219413)	UUGAGCCGCGCCAAUAUCACA	ppt-miR171a
4-72 (gnl|ti|993696673)	UUGAGCCGCGCCAAUAUCACA	ppt-miR171b
3-36	GCUACUUCGGCGGGACAAGAGA	ppt-miR903
2-70	GUUGGAAGCCUUCGUGGGA	ppt-miR1213
miR156	UGACAGAAGAGAGUGAGCAC	ppt-miR156b
miR160-1	UGCCUGGCUCCCUGUAUGCCA	ppt-miR160a
miR160-2	CGCCUGGCUCCCUGUAUGCCA	ppt-miR160b
miR160-3	CGCCUGGCUCCCUGCAUGCCA	ppt-miR160c
miR160-4	CGCCUGGCUCCCUGCAUGCCG	ppt-miR160d
miR165	UCGGACCAGGCUUCAUUCCCCU	ppt-miR166b
miR166	UCGGACCAGGCUUCAUUCCCU	ppt-miR166a
miR167	GGAAGCUGCCAGCAUGAUCCU	ppt-miR167
miR171-1	AGAUUGAGCCGCGCCAAUAUC	ppt-miR171c
miR171-2	UUGAGCCGGGCCAAUAUCACA	ppt-miR171d
miR319-1	UUGGACUGAAGGGAGCUCCA	ppt-miR319e
miR319-2	CUCGGACUGAAGGGAGCUCCC	ppt-miR319f
miR390-2 (gnl|ti|866247913)	AAGCUCAGGAGGGAUAGCGCC	ppt-miR390c
miR390-2 (gnl|ti|830400956)	AAGCUCAGGAGGGAUAGCGCC	ppt-miR390d
miR395	CUGAAGCGUUUGGGGGAAAGG	ppt-miR395
miR408	CUGCACUGCAUCUUCCCUGUGC	ppt-miR408
miR414	UCAUCCUCAUCAUCCUCGUCC	ppt-miR414
miR419	UGAUGAAUGAUGACGAUGUAU	ppt-miR419
miR473-1	CCUCUCCCUCAAAGGCUUCCA	ppt-miR473a
miR473-2	CCUCUCCCUCAAGGCUUCCA	ppt-miR473b
miR477 (PR1_miR477)	UUCUCCCUCAAAGGCUUCCAA	ppt-miR477a
miR477 (PR2_miR477)	UUCUCCCUCAAAGGCUUCCAA	ppt-miR477b
miR533-1	GAGCUGGCCAGGCUGUGAGGG	ppt-miR533a
miR533-2	GAGCUGUCCAGGCUGUGAGGG	ppt-miR533b
miR534-1	UAUGUCCAUUGCAGUUGCAUAC	ppt-miR534a
miR534-2	UAUGUCCAUUACAGUUGCAUAC	ppt-miR534b
miR535-1	UGACAACGAGAGAGAGCACGC	ppt-miR535d
miR535-2	UGACAUCGAGAGAGAGCACGC	ppt-miR535b
