# Supplementary figures and images for: Evidence for the rapid expansion of microRNA-mediated regulation in early land plant evolution
Source: BMC Plant Biol. 2007 Mar 14;7:13. doi: 10.1186/1471-2229-7-13 (PMC1838911; doi:10.1186/1471-2229-7-13)

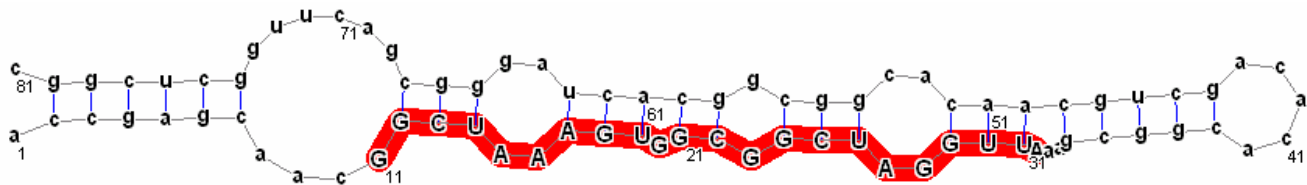

893327oryza (4-12 homolog)

Supplement: Additional file 4 — Precursor of a putative rice homolog of Physcomitrella miRNA 4–12. The reciprocal search with microHARVESTER using all Physcomitrella miRNAs without previously found homologs in other plants identified a putative homolog for miRNA 4–12 in rice. The corresponding precursor structure of this rice miRNA is depicted in this figure. [file 1471-2229-7-13-S4.pdf]
