# Supplementary material for: Evidence for the rapid expansion of microRNA-mediated regulation in early land plant evolution
Source: BMC Plant Biol. 2007 Mar 14;7:13. doi: 10.1186/1471-2229-7-13 (PMC1838911; doi:10.1186/1471-2229-7-13)
Supplement: Additional file 5 — Comparison of conserved plant miRNAs. In order to analyze conserved plant miRNA families all Physcomitrella miRNAs, as well as all plant miRNAs deposited in miRBase, were compared. miRNA sequences which were at least present in two different plant species are listed and the numbers of corresponding precursor sequences are provided. [file 1471-2229-7-13-S5.pdf]

Additional file 5. Comparison of conserved plant miRNAs.

miRNA family	Sequence	Dicots				Monocots				Moss
		ath	ptc	gma	mtr	zma	osa	sbi	sof	ppt
**MiRNA families not identified in *Physcomitrella***										
**159**	UUUGGAUUGAAGGGAGCUCUG					2	1	1	3	
	UUUGGAUUGAAGGGAGCUCUA	1	3	1						
	CUUGGAUUGAAGGGAGCUCCU		1			2		1	1	
	UUUGGAUUGAAGGGAGCUCUU	1								
	UUUGGAUUGAAGGGAGCUCCU	1								
	CUUGGGGUGAAGGGAGCUCCU		1							
	AUUGGAGUGAAGGGAGCUCGA		1							
	AUUGGAUUGAAGGGAGCUCCU						1			
	CUUGGAUUGAAGGGAGCUCUA						1			
	AUUGGAUUGAAGGGAGCUCCG						1			
	AUUGGAUUGAAGGGAGCUCCA						1			
	UUUGGAUUGAAAGGAGCUCUU								1	
**162**	UCGAUAAACCUCUGCAUCCAG	2	3				1			
	UCGAUAAGCCUCUGCAUCCAG						1			
	UCGAUAAACCUCUGCAUCCA					1				
**164**	UGGAGAAGCAGGGCACGUGCA	2	5			4	1	1		
	UGGAGAAGCAGGGCACGUGCU						1	1		
	UGGAGAAGCAGGGCACGUGCG	1								
	UGGAGAAGCAGGGCACAUGCU		1							
	UGGAGAAGCAGGGUACGUGCA						1			
	UGGAGAAGCAGGGCACGUGAG						1			
	UGGAGAAGCAGGACACGUGAG							1		
**168**	UCGCUUGGUGCAGAUCGGGAC					2	1	1	1	
	UCGCUUGGUGCAGGUCGGGAA	2	2	1						
	AGGCUUGGUGCAGCUCGGGAA						1			
	UCGCUUGGGCAGAUCGGGAC								1	
**169**	CAGCCAAGGAUGACUUGCCGA	1	3		1	2	1	1		
	CAGCCAAGGAUGACUUGCCGG	2	5	1		1	1	1		
	UAGCCAAGGAUGACUUGCCUG	7	5			3	1	4		
	UAGCCAAGGAUGACUUGCCUA		1			3	1	2		
	UAGCCAAGAAUGACUUGCCUA						1	1		
	UGAGCCAAGGAUGACUUGCCG	4	1							
	GAGCCAAGAAUGACUUGUCGG		1							
	AAGCCAAGGAUGACUUGCCUG		2							
	UCAGCCAAGGAUGACUUGCCG		1							
	UAGCCAAGGACGACUUGCCCA		5							
	GAGCCAAGAAUGACUUGCCGG		1							
	CAGCCAAGAAUGAUUUGCCGG		1							
	UAGCCAAGGACGACUUGCCUG		1							
	UAGCCAAGGACGACUUGCCUA		1							
	UAGCCAUGGAUGAAUUGCCUG		1							
	UAGCCAAGGAUGACUUGCCCA		2							
	UAGCCAAGGAUGACUUGCUCG		1							
	UAGCCAAGGAUGAAUUGCCGG						1			
	UAGCCAAGGACAAACUUGCCGG						1			
	UAGCCAAGGAUGACUUGCCGG						1			
	UAGCCAAGGAGACUGCCCAUG						1			
	UAGCCAAGGAGACUGCCUAUG					1				
	UAGCCAAGGAGACUGCCUACG					1				

miRNA family	Sequence	Dicots				Monocots				Moss
		ath	ptc	gma	mtr	zma	osa	sbi	sof	ppt
**MiRNA families not identified in *Physcomitrella***										
**169**	UCCGGCAAGUUGACCUUGGCU	1								
**393**	UCCAAAGGGAUCGCAUUGAUC	2	4		1		1	1		
	UCCAAAGGGAUCGCAUUGAUCU					1	1			
**394**	UUGGCAUUCUGUCCACCUCC	2	2			1	1	2		
**396**	UUCCACAGCUUUCUUGAACUG	1	2	1		2	1	2	1	
	UUCCACAGCUUUCUUGAACUU	1	3	1			1	1		
	UUCCACGGCUUUCUUGAACUG		1							
	UUCCACGGCUUUCUUGAACUU		1							
	UCCACAGGCUUUCUUGAACUG						1			
**397**	UCAUUGAGUGCAGCGUUGAUG	1	1				1			
	UCAUUGAGUGCAUCGUUGAUG	1								
	CCAUUGAGUGCAGCGUUGAUG		1							
	UCAUUGAGUGGAGCUUUGAUG		1							
	UUAUUGAGUGCAGCGUUGAUG						1			
**398**	UGUGUUCUCAGGUCACCCCUU	1	1	2			1			
	UGUGUUCUCAGGUCGCCCCUG		2				1			
	UGUGUUCUCAGGUCACCCCUG	2								
**399**	UGCCAAAGGAGAAUUGCCCUG		2			2	1	3		
	UGCCAAAGGAGAGUUGCCCUG	2				1	1	2		
	UGCCAAAGGAGAGCUGCCCUG				1	1	1	1		
	UGCCAAAGGAAAUUUGCCCCG					1	1	1		
	UGCCAAAGGAGAUUUGCCCAG				1		1	2		
	UGCCAAAGGAGAUUUGCCCGG	1	2							
	UGCCAAAGGAGAUUUGCCCCG	1	1							
	UGCCAAAGGAGAUUUGCCCUG	1			1					
	UGCCAAAGGAGAGUUGCCCUA		1				1			
	UGCCAAAGGAGAUUUGUCCGG		1							
	UGCCAAAGGAGAUUUGCUCAC		1							
	CGCCAAAGGAGAGUUGCCCUC		1							
	UGCCAAAGAAGAUUUGCCCCG		2							
	UGCCAAAGGAGAGUUUCCCUG		1							
	UGCCAAAGGAGAGCUGUCCUG					1				
	UGCCAAAGGAGAUUUGCCUCG	1								
	UGCCAAAGGAGACUUGCCCAG						1			
	UGCCAAAGGAGAGCUGCCCUA				1					
**403**	UUAGAUUCACGCACAAACUCG	1	2							
**413**	AUAGUUUCUCUUGUUCUGCAC	1								
	CUAGUUUCACUUGUUCUGCAC						1			
**415**	AACAGAGCAGAAACAGAACAU	1								
	AACAGAACAGAAGCAGAGCAG						1			
**416**	GGUUCGUACGUACACUGUUCA	1								
	UGUUCGUCCGUACACUGUUCA						1			
**417**	GAAGGUAGUGAAUUUGUUCGA	1								
	GAAUGUAGUGAAUUUGUUCCA						1			
**420**	UAAACUAAUCACGGAAAUGCA	1								
	UAAAUUAAUCACGGAAAUGAU						1			
**426**	UUUUGGAAAUUUGUCCUUACG	1								
	UUUUGGAAGUUUGUCCUUACG						1			

miRNA family	Sequence	Dicots				Monocots				Moss
		ath	ptc	gma	mtr	zma	osa	sbi	sof	ppt
**MiRNA families underrepresented in *Physcomitrella***										
**395**	CUGAAGUGUUUGGGGGAACUC	**3**	**9**							
	GUGAAGUGUUUGGGGGAACUC					**2**	**1**	**5**		
	GUGAAGUGUUUGGAGGAACUC					**1**	**1**			
	AUGAAGUGUUUGGGGGAACUC				**1**			**1**		
	GUGAAGUGCUUGGGGGAACUC						**1**			
	AUGAAGUGUUUGGAGGAACUC						**1**			
	GUGAAGUGUUUGGGGAAACUC						**1**			
	GUGAAUUGUUUGGGGGAACUC						**1**			
	GUGAAGUAUUUGGCGGAACUC						**1**			
	GUGAAGCGUUUGGGGGAAAUC						**1**			
	GUGAAGUGUUUGGGGGAUUCUC						**1**			
	UUGAAGUGUUUGGGGGAACUC				**1**					
	AUGAAGUGUUUGGGGGAACUU				**1**					
	AUGAAGUAUUUGGGGGAACUC				**1**					
	UUGAAGCGUUUGGGGGAACUC				**1**					
	CUGAAGGGUUUGGAGGAACUC		**1**							
	CUGAAGUGUUUGGGGGGACUC	**3**								
	CUGAAGCGUUUGGGGGAAAGG									**1**
**166**	UCGGACCAGGCUUCAUUCCCC	**7**	**13**	**2**	**1**	**1**	**1**			
	UCGGACCAGGCUUCAAUCCCU					**2**	**1**	**2**		
	UCGGACCAGGCUUCAUUCCUC					**2**	**1**	**1**		
	UCGGACCAGGCUUCAUUCCC					**8**		**4**		
	UCGGACCAGGCUUCAUUCCUU		**3**							
	UCGGACCAGGCUCCAUUCCUU		**1**							
	UCGAACCAGGCUUCAUUCCCC						**1**			
	UCGGAUCAGGCUUCAUUCCUC						**1**			
	UCGGACCAGGCUUCAUUCCCU						**1**			
	UCGGACCAGGCUUCAUUCCCU									**1**
**172**	AGAAUCUUGAUGAUGCUGCAU	**2**	**4**	**2**			**1**			
	GGAAUCUUGAUGAUGCUGCAU	**1**	**2**			**1**	**1**			
	AGAAUCUUGAUGAUGCUGCA					**4**		**3**		
	UGAAUCUUGAUGAUGCUGCAC						**1**	**1**		
	GCAGCACCAUUAAGAUUCAC	**1**								
	AGAAUCUUGAUGAUGCUGCAG	**2**								
	GGAAUCUUGAUGAUGCUGCA							**1**		
	GGAAUCUUGAUGAUGCUGCAG		**2**							
	AGAAUCCUGAUGAUGCUGCAA		**1**							
	AGAGAUUCUUGAUGAUGCUGAC									**1**
**167**	UGAAGCUGCCAGCAUGAUCUA	**2**	**4**	**2**		**4**	**1**	**2**		
	UGAAGCUGCCAGCAUGAUCUG	**1**	**1**			**5**	**1**	**5**	**2**	
	UUAAGCUGCCAGCAUGAUCUU	**1**								
	UGAAGCUGCCAGCAUGAUCUU		**2**							
	UGAAGCUGCCAACAUGAUCUG		**1**							
	GGAAGCUGCCAGCAUGAUCCU									**1**

miRNA family	Sequence	Dicots				Monocots				Moss
		ath	ptc	gma	mtr	zma	osa	sbi	sof	ppt
**MiRNA families overrepresented in *Physcomitrella***										
**390**	AAGCUCAGGAGGGAUAGCGCC	2	4				1			4
	GAGCUCAGGAGGGAUAGCGCC									1
	AGCUCAGGAGGGAUAGCGCC									1
**535**	UGACAACGAGAGAGAGCACGC						1			5
	UGACAACGAGAGAGAGUACGCU									n.p.
	UGACAUCGAGAGAGAGCACGC									1
**477**	AUCUCCCUCAGAGGCUUCCAA		2							
	UUCUCCCUCAAAGGCUUCCAA									2
	UCGGAAGCCUUUGUGGGAGAGGAA									n.p.
	UUCCUCUCCCACAAAGGCUUCCGA									n.p.
**160**	UGCCUGGCUCCCUGUAUGCCA	3	4	1	1	5	1	5		1
	UGCCUGGCUCCCUGAAUGCCA		2				1			
	UGCCUGGCUCCCUGUAUGCCG					1	1			
	UGCCUGGCUCCCUGGAUGCCA		1							
	UGCCUGGCUCCCUGCAUGCCA		1							
	CGCCUGGCUCCCUGCAUGCCG									1
	CGCCUGGCUCCCUGCAUGCCA									1
	CGCCUGGCUCCCUGUAUGCCA									1
	UCAUCCAGGGAGCCAGACAGA									1
**319**	UUGGACUGAAGGGAGCUCCC	2	4	2	1					
	UUGGACUGAAGGGUGCUCCC					4	1	1		
	UUGGACUGAAGGGAGCUCCU	1	4							
	UUGGGCUGAAGGGAGCUCCC		1							
	UUGGACUGAAAGGAGCUCCU			1						
	UUGGACUGAAGGGAGCUCCA									1
	CUCGGACUGAAGGGAGCUCCC									1
	CUUGGACUGAAGGGAGCUCC									2
	CUUGGACUGAAGGGAGCUCCC									2
	CUUGGACUGAAGGGAGCUUUUUUU									n.p.
**MiRNA families equally represented in *Physcomitrella***										
**408**	AUGCACUGCCUCUUCCCUGGC	1	1							
	CUGCACUGCCUCUUCCCUGGC					1	1		4	
	CUGCACUGACUCUUCCCUGGC								1	
	CUGCACUGCAUCUUCCCUGUGC									1
**171**	UGAUUGAGCCGUGCCAAUAUC		5			4	1	3		
	GAGGUGAGCCGAGCCAAUAUC					1	1	1		
	GUGAGCCGAACCAAUAUCACU					2	1	1		
	UGAUUGAGCCGCGCCAAUAUC	1					1			
	UUGAGCCGUGCCAAUAUCACG	2	2							
	UGAUUGAGCCGCGCCAAUAU					1				
	UUGAGCCGUGCCAAUAUCACA					1				
	AGAUUGAGCCGCGCCAAUAUC		2							1
	GGAUUGAGCCGCGCCAAUACU		1							
	UUGAGCCGUGCCAAUAUCAC					1				
	UGACUGAGCCGUGCCAAUAUC					1				
	UGAUUGAGUCGUGCCAAUAUC				1					
	GGAUUGAGCCGCGUCAAUAUC						1			
	AUGAGCCGAACCAAUAUCACU							1		

miRNA family	Sequence	Dicots				Monocots				Moss
		ath	ptc	gma	mtr	zma	osa	sbi	sof	ppt
**MiRNA families equally represented in *Physcomitrella***										
**171**	UUGAGCCGCGCCAAUAUCACA									**2**
	UUGAGCCGGGCCAAUAUCACA									**1**
**156**	UGACAGAAGAGAGUGAGCAC	**6**	**6**	**1**		**9**	**1**	**3**	**1**	**2**
	UGACAGAAGAGAGAGAGCACA			**1**	**1**	**1**	**1**	**1**		
	UUGACAGAAGAUAGAGAGCAC		**4**	**3**						
	UGACAGAAGAGAGCGAGCAC					**1**		**1**		
	UGACAGAAGAGAGGGAGCAC		**1**							
	CGACAGAAGAGAGUGAGCAUA						**1**			
	CGACAGAAGAGAGUGAGCACA	**1**								
	UUGACAGAAGAAAGAGAGCAC	**1**								
	UGACAGAAGAGAGUGAGCACAU									**n.p.**
**414**	UCAUCUUCAUCAUCAUCGUCA	**1**								
	UCAUCCUCAUCAUCAUCGUCC						**1**			
	UCAUCCUCAUCAUCCUCGUCC									**1**
**418**	UAAUGUGAUGAUGAACUGACC	**1**								
	UAAUGUGAUGAUGAAAUGACG						**1**			
	ACAUGUGAUGAAGAACUGACA									**1**
**419**	UUAUGAAUGCUGAGGAUGUUG	**1**								
	UGAUGAAUGCUGACGAUGUUG						**1**			
	UGAUGAAUGAUGACGAUGUAU									**1**
**165**	UCGGACCAGGCUUCAUCCCCC	**2**								
	UCGGACCAGGCUUCAUUCCCCU									**1**
**473**	ACUCUCCCUCAAGGCUUCCA		**1**							
	GCUCUCCCUCAGGGCUUCCA		**1**							
	CCUCUCCCUCAAAGGCUUCCA									**1**
	CCUCUCCCUCAAGGCUUCCA									**1**

MiRNA sequences which were at least present in two different plant species are listed. All previously reported *Physcomitrella* miRNAs and those identified in this study were included. MiRNA sequences from other plant species were retrieved from miRBase (version 8.2). Individual miRNAs present in one species are indicated by filled boxes. The numbers indicate the number of genes encoding this miRNA. ath: *Arabidopsis thaliana*, gma: *Glycine max*, mtr: *Medicago truncatula*, osa: *Oryza sativa*, ptc: *Populus trichocarpa*, ppt: *Physcomitrella patens*, sbi: *Sorghum bicolor*, sof: *Saccharum officinarum*, zma: *Zea mays*.

n.p.: no precursor identified.
