# Supplementary material for: Evidence for the rapid expansion of microRNA-mediated regulation in early land plant evolution
Source: BMC Plant Biol. 2007 Mar 14;7:13. doi: 10.1186/1471-2229-7-13 (PMC1838911; doi:10.1186/1471-2229-7-13)
Supplement: Additional file 6 — Sequence alignments of Physcomitrella miRNAs and their putative targets. The figure shows all sequence alignments between Physcomitrella miRNAs and their putative targets detected in a Physcomitrella EST database using the RNAhybrid program. [file 1471-2229-7-13-S6.pdf]

```
--------------------------------------------------------------
miRNA: 1-22 p_value: 2.76e-03 Energy: -32.4 kcal/mol
--------------------------------------------------------------
moss: T_1-22      Position: 483      Length: 938
Orientation: sense

            C      C          C
Target:      UUCCAGU CAAGUCCCAA
             |:||||: ||||||||||
miRNA:       AGGGUCG GUUCAGGGUU
            C      U          A
--------------------------------------------------------------
miRNA: 1-39 p_value: 1.05e-03 Energy: -32 kcal/mol
--------------------------------------------------------------
moss: T_1-39      Position: 319      Length: 1455
Orientation: sense

            G     U       G     C
Target:      GGUGA CCCGACG GAAAC
             ||||| ||||||| |||||
miRNA:       CCACU GGGCUGC CUUUG
                  U       A     C
--------------------------------------------------------------
miRNA: 1-63 p_value: 3.44e-04 Energy: -30.7 kcal/mol
--------------------------------------------------------------
moss: T1_1-63     Position: 279      Length: 549
Orientation: sense

             U      U          U
Target:       CUAAGU GUGCACAGCA
              |||||| ||||||||||
miRNA:        GAUUCA CACGUGUCGU
            AU      U          U
--------------------------------------------------------------
miRNA: 1-63 p_value: 6.45e-04 Energy: -30 kcal/mol
--------------------------------------------------------------
moss: T2_1-63     Position: 500      Length: 640
Orientation: sense

             C    U             G
Target:       CUAA UGGUGCACAGCAA
              |||| |:|||||||||||
miRNA:        GAUU AUCACGUGUCGUU
            AU    C
--------------------------------------------------------------
miRNA: 1-63 p_value: 5.29e-04 Energy: -32.4 kcal/mol
--------------------------------------------------------------
moss: T3_1-63     Position: 55       Length: 1201
Orientation: sense

            A  G               G
Target:      AC AGGUAGUGCACAGCG
             || |:||||||||||||:
miRNA:       UG UUCAUCACGUGUCGU
            A  A               U
---------------------------------------------------------------
```

```
--------------------------------------------------------------
miRNA: 2-28 p_value: 3.00e-05 Energy: -37.1 kcal/mol
--------------------------------------------------------------
moss: T_2-28      Position: 295      Length: 613
Orientation: sense

            G      G            U
Target:      GAUGCU AGAAUGGACAGC
             :||||| ||||||||||||
miRNA:       UUACGA UCUUACCUGUCG
            G      G            C
--------------------------------------------------------------
miRNA: 2-42 p_value: 0.00e+00 Energy: -53.2 kcal/mol
--------------------------------------------------------------
moss: T_2-42      Position: 767      Length: 1200
Orientation: sense

            C                         G
Target:      GCCUUAACCACUCGGCCAAAUUGAC
             |||||||||||||||||||||||||
miRNA:       CGGAAUUGGUGAGCCGGUUUAACUG

--------------------------------------------------------------
miRNA: 2-88 p_value: 3.50e-03 Energy: -30.7 kcal/mol
--------------------------------------------------------------
moss: T1_2-88     Position: 241      Length: 2507
Orientation: sense

             G              C   U
Target:       GUUCACUCUCUUCU UCG
              |:||||||||||| ||:
miRNA:        CGAGUGAGAGAAGA AGU
            CA              C
--------------------------------------------------------------
miRNA: 2-88 p_value: 1.10e-04 Energy: -38.5 kcal/mol
--------------------------------------------------------------
moss: T2_2-88     Position: 587      Length: 797
Orientation: sense

             A                 U
Target:       GCUCACUCUCUUCUGUC
              |||||||||||||||||
miRNA:        CGAGUGAGAGAAGACAG
            CA                 U
--------------------------------------------------------------
miRNA: 3-14 p_value: 1.50e-04 Energy: -36 kcal/mol
--------------------------------------------------------------
moss: T1_3-14     Position: 216      Length: 775
Orientation: sense

            G   U               A
Target:      AUU CUGUGCACUGCCUGG
             ||: |||||||||||||:|
miRNA:       UAG GACACGUGACGGAUC
            A   C               G
---------------------------------------------------------------
```

```
------------------------------------------------------------
miRNA: 3-14 p_value: 3.71e-03 Energy: -35.3 kcal/mol
------------------------------------------------------------
moss: T2_3-14     Position: 334      Length: 1992
Orientation: sense

             U    A              C
Target:       UCGU GUGCACUGUCUAGU
              |||: ||||||||:||||:
miRNA:        AGCG CACGUGACGGAUCG
            AU    A
------------------------------------------------------------
miRNA: 3-36 p_value: 0.00e+00 Energy: -48.8 kcal/mol
------------------------------------------------------------
moss: T_3-36      Position: 166      Length: 388
Orientation: sense

            G                     C
Target:      CUCUUGUCCCGCCGAAGUAGC
             |||||||||||||||||||||
miRNA:       GAGAACAGGGCGGCUUCAUCG
            A
------------------------------------------------------------
miRNA: 3-79 p_value: 1.67e-04 Energy: -37.6 kcal/mol
------------------------------------------------------------
moss: T_3-79      Position: 760      Length: 860
Orientation: sense

            C       C            A
Target:      UAUCCGG GAGCCGGACAGG
             :||||:| |||||:|||||:
miRNA:       GUAGGUC CUCGGUCUGUCU
            A       C
------------------------------------------------------------
miRNA: 3-91 p_value: 8.13e-04 Energy: -33.5 kcal/mol
------------------------------------------------------------
moss: T1_3-91     Position: 529      Length: 1226
Orientation: sense

            G    C              C
Target:      CCGG UGCAAGGACACAGC
             ||:| |:||||:|||||||
miRNA:       GGUC AUGUUCUUGUGUCG
            G    C
------------------------------------------------------------
miRNA: 3-91 p_value: 4.37e-04 Energy: -35.3 kcal/mol
------------------------------------------------------------
moss: T2_3-91     Position: 958      Length: 1433
Orientation: sense

             A           G     A
Target:       CAGGUACAAGAA ACAGC
              |||||||||||| |||||
miRNA:        GUCCAUGUUCUU UGUCG
            GG           G
------------------------------------------------------------
```

```
--------------------------------------------------------------
miRNA: 4-67 p_value: 1.90e-05 Energy: -37.6 kcal/mol
--------------------------------------------------------------
moss: T_4-67      Position: 206      Length: 751
Orientation: sense

             C     U              C
Target:       AGCGC GAGCUUGGCACGAU
              |||:| :|||||||||||||
miRNA:        UCGUG UUCGAACCGUGCUA
            UU     U
--------------------------------------------------------------
miRNA: 5-21 p_value: 9.00e-04 Energy: -29.5 kcal/mol
--------------------------------------------------------------
moss: T_5-21      Position: 281      Length: 1141
Orientation: sense

            A     A             C
Target:      GCUCC AAACAUUGACGAG
             ||:|| ||||||||||:||
miRNA:       CGGGG UUUGUAACUGUUC
                  A             U
--------------------------------------------------------------
miRNA: 5-33 p_value: 3.21e-04 Energy: -32.1 kcal/mol
--------------------------------------------------------------
moss: T_5-33      Position: 305      Length: 806
Orientation: sense

            G       U      G     U
Target:      GGCCUGU GAAACA CUCAA
             :|||||| |||||| |||||
miRNA:       UCGGACA CUUUGU GAGUU
                    U      G
--------------------------------------------------------------
miRNA: miR160-1/2 p_value: 5.70e-04 Energy: -36.5 kcal/mol
--------------------------------------------------------------
moss: T_miR160-1/2      Position: 600      Length: 778
Orientation: sense

            C     GC           A U
Target:      UGGCA  CGGGGAGCCAG C
             |||||  |:||||||||| |
miRNA:       ACCGU  GUCCCUCGGUC G
                  AU           C U
--------------------------------------------------------------
miRNA: miR160-1/2/3/4   p_value: 1.90e-05 Energy: -50.4 kcal/mol
--------------------------------------------------------------
moss: T_miR160-1/2/3/4  Position: 1924     Length: 2844
Orientation: sense

            U                     U
Target:      UGGCAUGCAGGGGGCCAGGCA
             ||||||:|||||:||||||||
miRNA:       ACCGUAUGUCCCUCGGUCCGU
--------------------------------------------------------------
```

```
--------------------------------------------------------------
miRNA: miR160-2/3/4     p_value: 6.27e-04 Energy: -37.3 kcal/mol
--------------------------------------------------------------
moss: T_miR160-2/3/4    Position: 158      Length: 1032
Orientation: sense

             A   G       C       G
Target:       GCA GCAGGGA CCAGGCG
              ||| :|||||| |||||||
miRNA:        CGU UGUCCCU GGUCCGC
            AC   A       C
--------------------------------------------------------------
miRNA: miR160-3   p_value: 5.86e-04 Energy: -38.9 kcal/mol
--------------------------------------------------------------
moss: T_miR160-3  Position: 389      Length: 1027
Orientation: sense

            G  GC  G            A
Target:      GG  UG GGGGAGCCAGGC
             ||  || :|||||||||||
miRNA:       CC  AC UCCCUCGGUCCG
            A  GU  G            C
--------------------------------------------------------------
miRNA: miR166     p_value: 8.63e-04 Energy: -38.5 kcal/mol
--------------------------------------------------------------
moss: T_miR166    Position: 1074     Length: 3591
Orientation: sense

             U  U                A
Target:       GG AUGAAGCCUGGUCCGG
              || |||||||||||||||:
miRNA:        CC UACUUCGGACCAGGCU
            UC  U
--------------------------------------------------------------
miRNA: miR167     p_value: 1.95e-04 Energy: -37.2 kcal/mol
--------------------------------------------------------------
moss: T1_miR167   Position: 916      Length: 2017
Orientation: sense

            U        C            G
Target:      AGGAUUGU UUGGCAGCUUCC
             |||||::| :|||||||||||
miRNA:       UCCUAGUA GACCGUCGAAGG
                     C
--------------------------------------------------------------
miRNA: miR167     p_value: 2.20e-05 Energy: -37.7 kcal/mol
--------------------------------------------------------------
moss: T2_miR167   Position: 328      Length: 750
Orientation: sense

             U   G               A
Target:       GGU GUGCUGGCAGCUUCU
              |:| :|||||||||||||:
miRNA:        CUA UACGACCGUCGAAGG
            UC   G
--------------------------------------------------------------
```

```
-------------------------------------------------------------
miRNA: miR171-1   p_value: 1.94e-04 Energy: -32.5 kcal/mol
-------------------------------------------------------------
moss: T_miR171-1  Position: 224      Length: 617
Orientation: sense

             C    U              U
Target:       UGUU GUGCGGCUCAGUCU
              |:|| |:|||||||:|||
miRNA:        AUAA CGCGCCGAGUUAGA
            CU    C
-------------------------------------------------------------
miRNA: miR319-1   p_value: 3.67e-04 Energy: -35.3 kcal/mol
-------------------------------------------------------------
moss: T_miR319-1  Position: 904      Length: 1250
Orientation: sense

            G A                    A
Target:      G GGCUUCCUUCAGUCCAG
             | :|||:|||||||||||:
miRNA:       C UCGAGGGAAGUCAGGUU
            A C
-------------------------------------------------------------
miRNA: miR408     p_value: 1.60e-05 Energy: -40.5 kcal/mol
-------------------------------------------------------------
moss: T1_miR408   Position: 28       Length: 718
Orientation: sense

            G  U     GG           C
Target:      GC CGGGG  GAUGCGGUGCA
             || |:|||  |||||:|||||
miRNA:       CG GUCCC  CUACGUCACGU
               U     UU           C
-------------------------------------------------------------
miRNA: miR408     p_value: 0.00e+00 Energy: -49.6 kcal/mol
-------------------------------------------------------------
moss: T2_miR408   Position: 118      Length: 760
Orientation: sense

            G                      U
Target:      GCACAGGGAAGAUGCAGUGCAG
             ||||||||||||||||||||||
miRNA:       CGUGUCCCUUCUACGUCACGUC
-------------------------------------------------------------
miRNA: miR408     p_value: 5.00e-06 Energy: -43.1 kcal/mol
-------------------------------------------------------------
moss: T3_miR408   Position: 222      Length: 722
Orientation: sense

            G  U                  A
Target:      GC CAGGGAAGAUGCAGUGCA
             || ||||||||||||||||||
miRNA:       CG GUCCCUUCUACGUCACGU
               U                  C
-------------------------------------------------------------
```

```
-------------------------------------------------------------
miRNA: miR408     p_value: 2.70e-05 Energy: -39.2 kcal/mol
-------------------------------------------------------------
moss: T4_miR408   Position: 50       Length: 705
Orientation: sense

            U        GC           C
Target:      GUGCGGGG  GAUGCGGUGCA
             |::|:|||  |||||:|||||
miRNA:       CGUGUCCC  CUACGUCACGU
                     UU           C
-------------------------------------------------------------
miRNA: miR408     p_value: 0.00e+00 Energy: -49.6 kcal/mol
-------------------------------------------------------------
moss: T5_miR408   Position: 159      Length: 805
Orientation: sense

            G                      U
Target:      GCACAGGGAAGAUGCAGUGCAG
             ||||||||||||||||||||||
miRNA:       CGUGUCCCUUCUACGUCACGUC
-------------------------------------------------------------
miRNA: miR414     p_value: 3.22e-04 Energy: -33 kcal/mol
-------------------------------------------------------------
moss: T1_miR414   Position: 296      Length: 1274
Orientation: sense

            A        A           A
Target:      GGUGGGGA GAUGAGGAUGA
             |::|:||| |||||||||||
miRNA:       CUGCUCCU CUACUCCUACU
            C        A
-------------------------------------------------------------
miRNA: miR414     p_value: 2.85e-04 Energy: -33.4 kcal/mol
-------------------------------------------------------------
moss: T2_miR414   Position: 205      Length: 1323
Orientation: sense

            A  UG U            G U
Target:      GG  G GGAUGAUGAGGA G
             ||  | |||||||||||| |
miRNA:       CC  C CCUACUACUCCU C
               UG U            A U
-------------------------------------------------------------
miRNA: miR414     p_value: 1.71e-04 Energy: -33.9 kcal/mol
-------------------------------------------------------------
moss: T3_miR414   Position: 344      Length: 1087
Orientation: sense

            A               A    U
Target:      GAUGAGGAUGAUGAG AUGG
             ||:|||||||||||| |||:
miRNA:       CUGCUCCUACUACUC UACU
            C               C
-------------------------------------------------------------
```

```
-------------------------------------------------------------
miRNA: miR414     p_value: 7.70e-05 Energy: -34.8 kcal/mol
-------------------------------------------------------------
moss: T4_miR414   Position: 265      Length: 851
Orientation: sense

            C  UG U           U   G
Target:      GG  G GGAUGAUGAGG UGG
             ||  | ||||||||||| ||:
miRNA:       CC  C CCUACUACUCC ACU
               UG U           U
-------------------------------------------------------------
miRNA: miR414     p_value: 2.00e-06 Energy: -41.6 kcal/mol
-------------------------------------------------------------
moss: T5_miR414   Position: 201      Length: 655
Orientation: sense

            U                    A
Target:      GAUGAGGAUGAUGAGGAUGA
             ||:|||||||||||||||||
miRNA:       CUGCUCCUACUACUCCUACU
            C
-------------------------------------------------------------
miRNA: miR414     p_value: 2.57e-04 Energy: -32.7 kcal/mol
-------------------------------------------------------------
moss: T6_miR414   Position: 267      Length: 997
Orientation: sense

            C     C  G           U
Target:      GACGA GA GAUGAGGAUGA
             ||||| || |||||||||||
miRNA:       CUGCU CU CUACUCCUACU
            C     C  A
-------------------------------------------------------------
miRNA: miR414     p_value: 2.18e-04 Energy: -32.9 kcal/mol
-------------------------------------------------------------
moss: T7_miR414   Position: 413      Length: 949
Orientation: sense

            C     C           C  G
Target:      GACGA GAUGAUGAGGA GA
             ||||| ||||||||||| ||
miRNA:       CUGCU CUACUACUCCU CU
            C     C           A
-------------------------------------------------------------
miRNA: miR414     p_value: 2.80e-05 Energy: -36.1 kcal/mol
-------------------------------------------------------------
moss: T8_miR414   Position: 444      Length: 664
Orientation: sense

            U     A              U
Target:      GACGA GGUGAUGAGGAUGA
             ||||| |:||||||||||||
miRNA:       CUGCU CUACUACUCCUACU
            C     C
-------------------------------------------------------------
```

```
-------------------------------------------------------------
miRNA: miR414     p_value: 1.17e-04 Energy: -33.7 kcal/mol
-------------------------------------------------------------
moss: T9_miR414   Position: 575      Length: 803
Orientation: sense

            C  A        A        A
Target:      GA GAGGAUGA GAGGAUGA
             || |||||||| ||||||||
miRNA:       CU CUCCUACU CUCCUACU
            C  G        A
-------------------------------------------------------------
miRNA: miR414     p_value: 2.82e-04 Energy: -32.9 kcal/mol
-------------------------------------------------------------
moss: T10_miR414  Position: 488      Length: 1128
Orientation: sense

            A      A     A        G
Target:      GGAUGA GGUGA GAGGAUGA
             |||:|| |:||| ||||||||
miRNA:       CCUGCU CUACU CUCCUACU
                   C     A
-------------------------------------------------------------
miRNA: miR414     p_value: 6.50e-05 Energy: -37.4 kcal/mol
-------------------------------------------------------------
moss: T11_miR414  Position: 771      Length: 1585
Orientation: sense

            U               U     U
Target:      GGAUGAGGAUGAUGA GAUGA
             |||:||||||||||| |||||
miRNA:       CCUGCUCCUACUACU CUACU
                            C
-------------------------------------------------------------
miRNA: miR414     p_value: 6.00e-06 Energy: -37 kcal/mol
-------------------------------------------------------------
moss: T12_miR414  Position: 435      Length: 805
Orientation: sense

            U       CC          U
Target:      GGCGAGG  GAUGAGGAUGA
             |:|||||  |||||||||||
miRNA:       CUGCUCC  CUACUCCUACU
            C       UA
-------------------------------------------------------------
miRNA: miR418     p_value: 0.00e+00 Energy: -40.2 kcal/mol
-------------------------------------------------------------
moss: T_miR418    Position: 655      Length: 1066
Orientation: sense

            A                     G
Target:      UGUCAGUUCUUCAUCACAUGU
             |||||||||||||||||||||
miRNA:       ACAGUCAAGAAGUAGUGUACA

-------------------------------------------------------------
```

```
--------------------------------------------------------------
miRNA: miR419     p_value: 9.35e-04 Energy: -27.8 kcal/mol
--------------------------------------------------------------
moss: T_miR419    Position: 1375     Length: 1514
Orientation: sense

            G        A            C
Target:      GUAUGUUG CAUCGUUCAUCA
             :||::|:| ||||:|||||||
miRNA:       UAUGUAGC GUAGUAAGUAGU
                     A
--------------------------------------------------------------
miRNA: miR473-2   p_value: 2.46e-04 Energy: -35.1 kcal/mol
--------------------------------------------------------------
moss: T1_miR473-2 Position: 377      Length: 823
Orientation: sense

             A                 U
Target:       GGAGCUUUGAGGGAGGG
              |:|||:|||||||||:|
miRNA:        CUUCGGAACUCCCUCUC
            AC                 C
--------------------------------------------------------------
miRNA: miR473-2   p_value: 2.70e-05 Energy: -38.8 kcal/mol
--------------------------------------------------------------
moss: T2_miR473-2 Position: 533      Length: 556
Orientation: sense

            A UU                C
Target:      G  AGCCUUGAGGGAGAGG
             |  ||||||||||||||||
miRNA:       C  UCGGAACUCCCUCUCC
            A CU
--------------------------------------------------------------
miRNA: miR473-2   p_value: 5.44e-04 Energy: -35 kcal/mol
--------------------------------------------------------------
moss: T3_miR473-2 Position: 1354     Length: 1402
Orientation: sense

             A     AG           A
Target:       GAAGC  UGAGGGAGAGG
              |||||  |||||||||||
miRNA:        CUUCG  ACUCCCUCUCC
            AC     GA
--------------------------------------------------------------
miRNA: miR477     p_value: 6.00e-06 Energy: -43.8 kcal/mol
--------------------------------------------------------------
moss: T1_miR477   Position: 173      Length: 664
Orientation: sense

            U                     A
Target:      UUGGGAGCCUUUGAGGGAGAA
             ||||:||||||||||||||||
miRNA:       AACCUUCGGAAACUCCCUCUU
--------------------------------------------------------------
```

```
--------------------------------------------------------------
miRNA: miR477     p_value: 5.60e-05 Energy: -38.1 kcal/mol
--------------------------------------------------------------
moss: T2_miR477   Position: 14       Length: 656
Orientation: sense

             A   U              U
Target:       GGA GCCUUUGAGGGAGA
              ||| ||||||||||||||
miRNA:        CCU CGGAAACUCCCUCU
            AA   U              U
--------------------------------------------------------------
miRNA: miR477     p_value: 2.00e-06 Energy: -43 kcal/mol
--------------------------------------------------------------
moss: T3_miR477   Position: 419      Length: 1660
Orientation: sense

            G                   U
Target:      UGGAGGCCUUUGAGGGAGA
             ||||:||||||||||||||
miRNA:       ACCUUCGGAAACUCCCUCU
            A                   U
--------------------------------------------------------------
miRNA: miR477     p_value: 1.74e-04 Energy: -36.2 kcal/mol
--------------------------------------------------------------
moss: T4_miR477   Position: 182      Length: 833
Orientation: sense

             G    U              G
Target:       GGAA CCUUUGAGGGAGAA
              |||| ||||||||||||||
miRNA:        CCUU GGAAACUCCCUCUU
            AA    C
--------------------------------------------------------------
miRNA: miR477     p_value: 5.83e-04 Energy: -31.7 kcal/mol
--------------------------------------------------------------
moss: T5_miR477   Position: 142      Length: 543
Orientation: sense

             G     AG           C
Target:       GGAGG  UUUGAGGGAGG
              |||:|  ||||||||||:
miRNA:        CCUUC  AAACUCCCUCU
            AA     GG           U
---------------------------------------------------------------
miRNA: miR477     p_value: 1.90e-05 Energy: -37.9 kcal/mol
---------------------------------------------------------------
moss: T6_miR477   Position: 1257     Length: 1410
Orientation: sense

             G   U              U
Target:       GGA GCCUUUGAGGGAGA
              ||| ||||||||||||||
miRNA:        CCU CGGAAACUCCCUCU
            AA   U              U
---------------------------------------------------------------
```

```
--------------------------------------------------------------
miRNA: miR477, miR473-1 p_value: 6.87e-04 Energy: -32 kcal/mol
--------------------------------------------------------------
moss: T_miR473-1/miR477 Position: 533      Length: 668
Orientation: sense

             A     A A           A
Target:       GGAAG C UUGAGGGAGAG
              ||||| | ||||||||||:
miRNA:        CCUUC G AACUCCCUCUU
            AA     G A
--------------------------------------------------------------
miRNA: miR533-2   p_value: 2.81e-04 Energy: -37.4 kcal/mol
--------------------------------------------------------------
moss: T_miR533-2  Position: 486      Length: 830
Orientation: sense

            U     CG               A
Target:      UCUUC  AGCCUGGACAGCUU
             :|:||  |||||||||||||:
miRNA:       GGGAG  UCGGACCUGUCGAG
                  UG
--------------------------------------------------------------
miRNA: miR534-1   p_value: 4.56e-04 Energy: -31.5 kcal/mol
--------------------------------------------------------------
moss: T1_miR534-1 Position: 40       Length: 690
Orientation: sense

             G            C      U
Target:       AUGCAAUUGCAA GGACAU
              ||||||:||||| ||||||
miRNA:        UACGUUGACGUU CCUGUA
            CA            A      U
--------------------------------------------------------------
miRNA: miR534-1   p_value: 3.00e-06 Energy: -40.8 kcal/mol
--------------------------------------------------------------
moss: T2_miR534-1 Position: 150      Length: 719
Orientation: sense

            U                      G
Target:      UGUGCAACUGCAAUGGACAUA
             |:|||||||||||||||||||
miRNA:       AUACGUUGACGUUACCUGUAU
            C
--------------------------------------------------------------
```
